# Multifunctional Hydrogels with Reversible 3D Ordered Macroporous Structures

**DOI:** 10.1002/advs.201500069

**Published:** 2015-03-26

**Authors:** Hongkun He, Saadyah Averick, Pratiti Mandal, Hangjun Ding, Sipei Li, Jeff Gelb, Naomi Kotwal, Arno Merkle, Shawn Litster, Krzysztof Matyjaszewski

**Affiliations:** ^1^ Center for Macromolecular EngineeringDepartment of ChemistryCarnegie Mellon UniversityPittsburghPA15213USA; ^2^ Department of Mechanical EngineeringCarnegie Mellon UniversityPittsburghPA15213USA; ^3^ School of Materials Science and EngineeringUniversity of Science and Technology Beijing30 Xueyuan RoadBeijing100083P. R. China; ^4^ Carl Zeiss X‐Ray MicroscopyPleasantonCA94588USA

**Keywords:** colloidal crystals, functional materials, hydrogels, porous polymer, shape memory

## Abstract

**Three‐dimensionally ordered macroporous (3DOM) hydrogels** prepared by colloidal crystals templating display highly reversible shape memory properties, as confirmed by indirect electron microscopy imaging of their inverse replicas and direct nanoscale resolution X‐ray microscopy imaging of the hydrated hydrogels. Modifications of functional groups in the 3DOM hydrogels result in various materials with programmed properties for a wide range of applications.

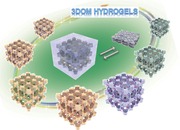

Hydrogels are synthetic or natural polymer networks crosslinked either chemically or physically to maintain 3D structures that are able to absorb and retain large amounts of water.^1^ Porous biocompatible hydrogels combine porosity with biocompatibility and play an important role in various biomedical applications, such as scaffold materials in tissue engineering, cell transplantation, and regenerative medicine.^2^ The properties of porous hydrogels are substantially influenced by the porosity related parameters, including surface area, pore size, pore interconnectivity, interfacial properties, etc.^3^ Therefore, rational design of hydrogels with desired structures and properties is an essential requirement for advanced technological applications. The properties can be additionally programmed by modification of functional porous hydrogels.

Macroporous hydrogels prepared by colloidal crystal templating have attracted much attention in recent years due to their unique structural features, holding immense promise as responsive materials for detection,^4^ scaffolds for tissue engineering,^5^ and absorbents for CO_2_ capture.^6^ The colloidal crystal templating is a facile and effective method to construct 3D ordered macroporous (3DOM) structures with interconnected pores and well‐controlled pore size.^7^ However, the porous structures of 3DOM hydrogels have not been definitively determined in many cases, which is largely due to the difficulty in morphological characterizations, and this has posed an obstacle to further precise studies.

Herein, we analyze the reversible 3DOM porous structures in colloidal crystal templated macroporous hydrogels through two approaches: one procedure is electron microscopy imaging of their inverse replicas and the other is noninvasive and nondestructive nanoscale resolution X‐ray microscopy (nano‐XRM) imaging of the hydrated hydrogels. Thus, one focus of this work is the development of two novel methods for the characterization of 3DOM structures in hydrogels. This work also demonstrates for the first time the intrinsic shape memory properties of 3DOM hydrogels. The additional aim of this work is further modification of functional 3DOM hydrogels. The properties of such structurally tailored and engineered macromolecular (*stem*) gels can be programmed, depending on the applied chemistry. Thus, the functional hydrogels represent a robust platform for constructing well‐defined functional materials with preselected properties targeting a wide range of applications.

The preparation of 3DOM hydrogels is schematically shown in **Figure** **1**. Uniform poly(methyl methacrylate) (PMMA) colloidal spheres with average diameter of 286 nm were synthesized by surfactant free emulsion polymerization and assembled to face centered cubic crystalline structure by centrifugation (Supporting Information, Figure S1). An aqueous solution of a mixture of monomer (i.e., poly(ethylene oxide) methacrylate (PEOMA)) and crosslinker (i.e., poly(ethylene oxide) dimethacrylate (PEODMA)) was infiltrated into the voids between the stacked PMMA spheres by capillary force, which should reduce the defects generated in the porous structure compared to infiltration under partial vacuum.[[qv: 6b]] Thermal‐initiated free radical copolymerization (FRP) of the monomer and crosslinker formed a crosslinked network (Figure 1a). The colloidal crystal template was then removed by washing with acetone to generate the desired porous structure. The colloidal crystal templating method for the preparation of macroporous hydrogels is as simple as other techniques, including freeze‐drying, porogenation, microemulsion formation, etc. The size of the macropores can be easily controlled by tuning the diameter of colloidal crystal templates by varying the synthetic conditions used for the preparation of the latex colloidal spheres.^8^

**Figure 1:**
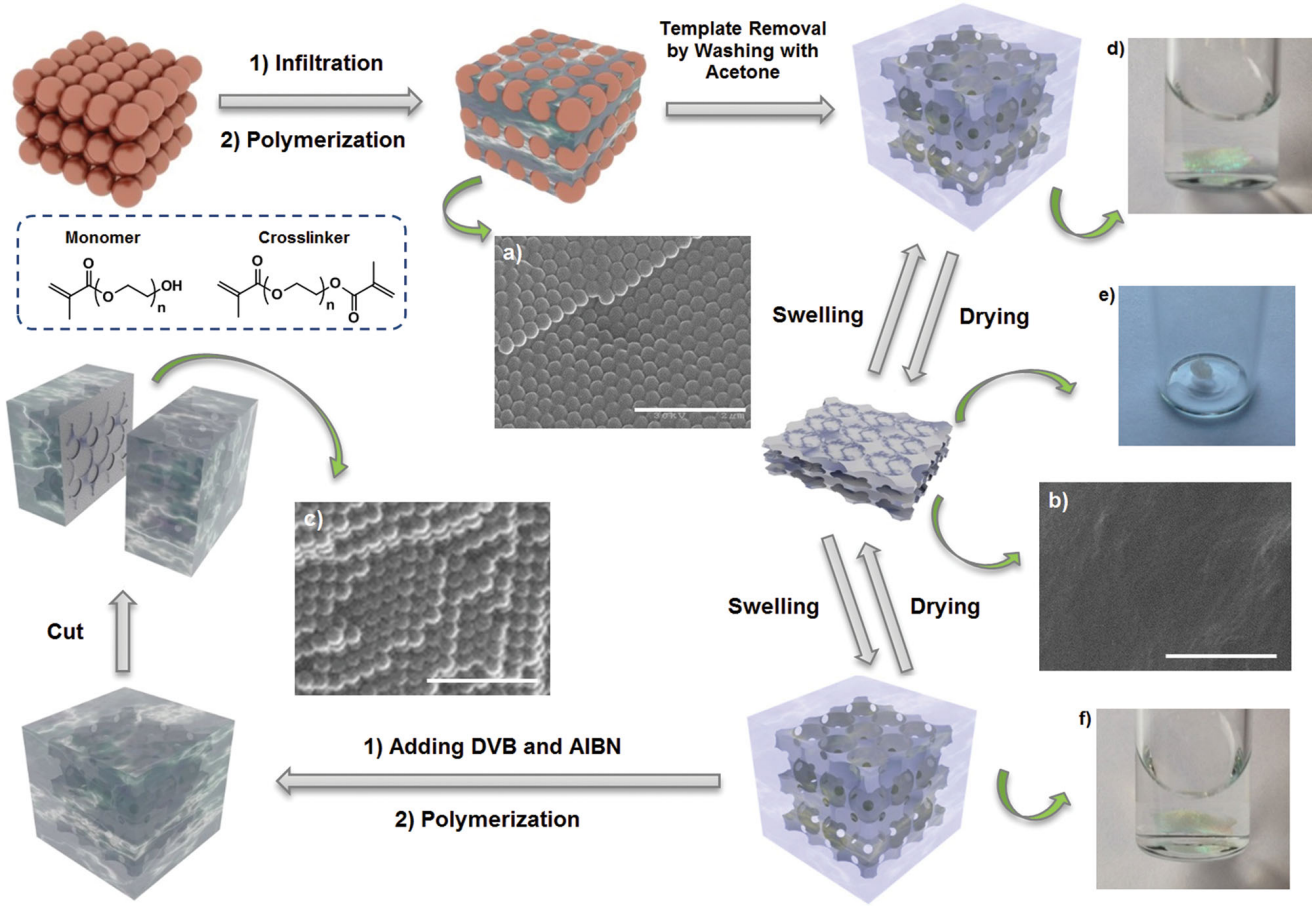
Preparation and characterizations of 3DOM hydrogels. Scale bar: 2 μm.

The structure of the resulting hydrogels was tentatively characterized by scanning electron microscopy (SEM); however, no pores were observed in the SEM images (Figure 1b). Nevertheless, the brilliant colors originating from Bragg diffraction of the hydrogels, when they were placed in solvents, indicated the presence of an ordered macroporous structure (Figure 1d–f). Thus, it was conceivable that the pores in the hydrogels collapsed during drying, prior to the SEM measurements.

In order to prevent collapse of macropores, more rigid water‐soluble monomers (i.e., methacrylates containing quaternary ammonium groups, see Supporting Information, Figure S2) were used, and the 3DOM hydrogels were synthesized by controlled radical polymerization (i.e., atom transfer radical polymerization (ATRP), see Supporting Information, Figure S3),^9^ but still no macropores were observed in the SEM images of the resulting hydrogels. It should be noted that the mechanical properties of the hydrogels can be influenced by the properties of both crosslinker and monomer as well as their ratio^10^ (Supporting Information, Figure S4). This allows the macropores to be persevered in hydrogels that are sufficiently strong, i.e., with very high content of rigid monomer/crosslinker (Supporting Information, Figure S5). Since collapse of macropores was still a major problem for soft hydrogels, new approaches were required to characterize the porous structure of soft 3DOM hydrogels.

A facile method was developed to resolve this problem and indirectly characterize the 3DOM structure by SEM. The porous hydrogels were soaked in an acetone solution of a crosslinker (i.e., divinylbenzene (DVB)), which was then thermally polymerized to form arrays of crosslinked DVB spheres inside the macropores of the hydrogels (Figure 1). The resulting composites were mechanically stable under vacuum because of the rigid crosslinked DVB networks, which were inverse replicas of the initial 3DOM structures. The SEM images of the section surfaces of the composites displayed ordered sphere arrays (Figure 1c), revealing the shape of the pores in the original 3DOM structures in the swollen hydrogels. This method was also successfully applied to characterize the 3DOM hydrogels with various crosslinking densities (10%, 30%, 50%, 70%, 90%, and 100%) (Supporting Information, Figure S6) and with different compositions (Supporting Information, Figure S7).

The macroporous structures of the 3DOM hydrogels were retained and highly reversible through repeated collapse and reformation cycles. The original 3DOM structures stayed intact as the 3DOM hydrogels were repeatedly dried under vacuum and then swollen by solvents for ten cycles. This was confirmed by the ordered sphere arrays in the SEM images of their inverse replicas (Supporting Information, Figure S6). This demonstrated the intrinsic reversible shape memory nature of the 3DOM structures in the hydrogels.

In addition, a direct approach was employed to resolve the 3DOM structure in the presence of water by nano‐XRM in Zernike phase contrast mode.^11^ The 3D nano‐XRM images (**Figure** **2** and Supporting Information, Figures S8–S10) and movies (Supporting Information, Movies S1–S3) displayed orthogonal virtual slices through the reconstructed volume, the surface view of a segmented cropped volume, and a volume rendering of the pore phase, which had a maximum spatial resolution of 50 nm (16 nm voxels).^12^ It revealed the 3D distributed porous structures with interconnected windows in the swollen samples. This is a nondestructive way to visualize the porous structures of swollen hydrogels in ambient conditions without requiring vacuum or pretreatment of samples.

**Figure 2:**
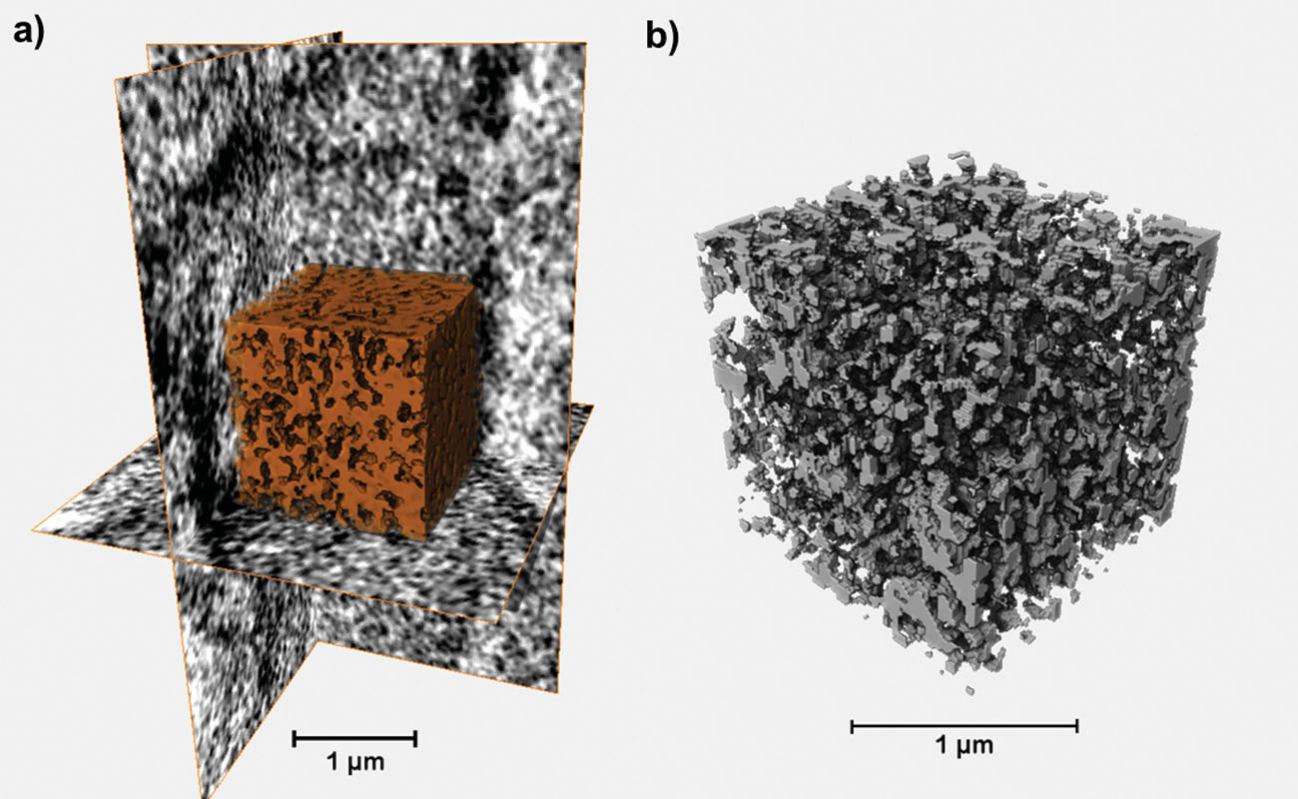
3D nano‐XRM images of trypsin immobilized 3DOM hydrogels soaked in water: A) orthogonal raw tomography slices through the reconstructed volume along with the surface view of a segmented cropped volume (the dark phase represents the pores), and B) volume rendering of the pore phase of the cropped volume.

These well‐defined 3DOM hydrogels can serve as a versatile platform for the preparation of a variety of functional materials. The presence of surface functional groups on the pores of the 3DOM hydrogels, originating from the functional monomers, permitted to design materials with new properties by further chemical modifications of the accessible functionalities via grafting with functional organic compounds and polymers. The functional hydrogels can be considered as *stem* (structurally tailored and engineered macromolecular) gels (by analogy with stem cells) that can evolve into materials with final properties and function that can be programmed, depending on the chemistry applied for modification. By the esterification of the hydroxyl groups of the 3DOM hydrogels with dodecanoyl chloride, the surfaces of hydrogels modified with the long alkyl chains became hydrophobic. The alternation of the hydrophilicity/hydrophobicity was verified by water contact angle measurements. Water droplet was absorbed by the pristine hydrogels, while it stayed on the surface of the modified hydrogels (**Figure** **3**A). A condensation reaction between the hydroxyl groups on the 3DOM hydrogels with rhodamine B afforded fluorescent hydrogels which could become fluorescent under UV light (Figure 3B). The hydroxyl groups of the 3DOM hydrogels were also converted to carboxylic acid groups by reacting with succinic anhydride and then polyaniline was generated through in situ oxidative polymerization of infused aniline using ammonium persulfate (Supporting Information, Figures S11–S13). The resulting 3DOM hydrogel/polyaniline composites were electronically conductive, enabling them to connect a circuit and light a light‐emitting diode (LED) lamp (Figure 3C).

**Figure 3:**
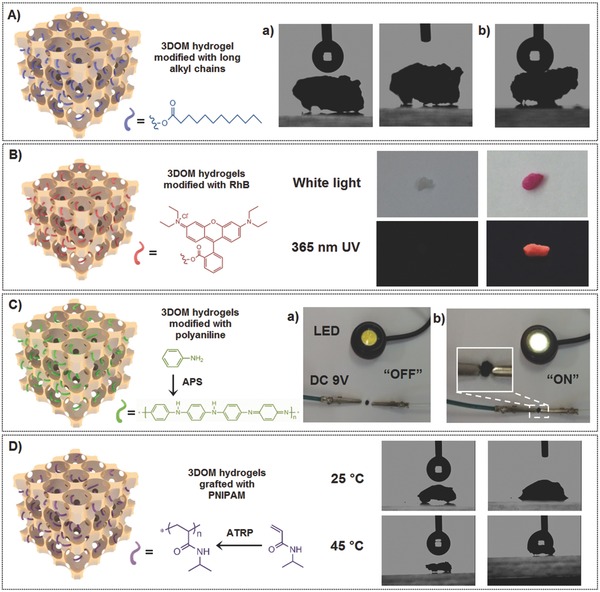
A) 3DOM hydrogel modified with long alkyl chains: the change of hydrophilicity/hydrophobicity was visualized by placing a drop of water on a) 3DOM hydrogel containing hydroxyl groups (immediate penetration of the water into the sample occurred and no drop is visible) and b) long alkyl chains modified 3DOM hydrogel (the water droplet stayed on top of the sample). B) 3DOM hydrogels modified with RhB: a comparison of 3DOM hydrogels containing hydroxyl groups before (left column) and after (right column) conjugation with RhB when exposed to white light (top row) or a UV source (bottom row). C) 3DOM hydrogels modified with polyaniline: photos of a LED circuit a) without and b) with connection of polyaniline modified 3DOM hydrogels. D) 3DOM hydrogels grafted with PNIPAM: change of hydrophilicity/hydrophobicity visualized by placing a drop of water on PNIPAM grafted 3DOM hydrogels at 25 and 45 °C (immediate penetration of the water into the sample occurred at 25 °C and no drop is visible; while the water droplet stayed on top of the sample at 45 °C).

In addition, ATRP initiators were anchored onto the surface of the pores by reaction between hydroxyl groups of the 3DOM hydrogels and α‐bromoisobutyryl bromide. Poly(*N*‐isopropylacrylamide) (PNIPAM) was then grafted from the pore walls by ATRP (Supporting Information, Figure S14) and the resulting copolymer showed temperature‐responsive surface properties due to the lower critical solution temperature (LCST) of PNIPAM (32 °C). As shown in Figure 3D, the PNIPAM grafted 3DOM hydrogels absorbed the water droplet quickly at 25 °C, whereas they became hydrophobic and repelled the water droplet at 45 °C.

The 3DOM hydrogels modified with bioactive species were useful for digestion or separation of bio(macro)molecules. For instance, trypsin was grafted onto the pores of the 3DOM hydrogels by the reaction of trypsin amino group with carboxylic acid groups from the hydrogels. The resulting 3DOM hydrogels with pore immobilized trypsin displayed bioactivity for tryptic digestion of bovine serum albumin (BSA) (**Figure** **4**b) and *N‐*α‐benzoyl‐l‐arginine *p*‐nitroanilide (BAPNA) (Supporting Information, Figures S16 and S17). While the use of enzyme immobilized inverse opals as heterogeneous biocatalysis has been demonstrated in previous reports,^13^ the present work allows fabrication of a novel column with trypsin‐immobilized packing (Figure 4a), which displays high performance in online hydrolysis using *N*‐α‐benzoyl‐l‐arginine ethyl ester hydrochloride (BAEE) as the substrate (Figure 4c and Supporting Information, Figures S18–S26). The advantages are that the 3DOM structures can provide large surface area and reduced mass transfer resistance, making the enzyme‐immobilized 3DOM hydrogels efficient biocatalyst for column filtration/separation.^14^

**Figure 4:**
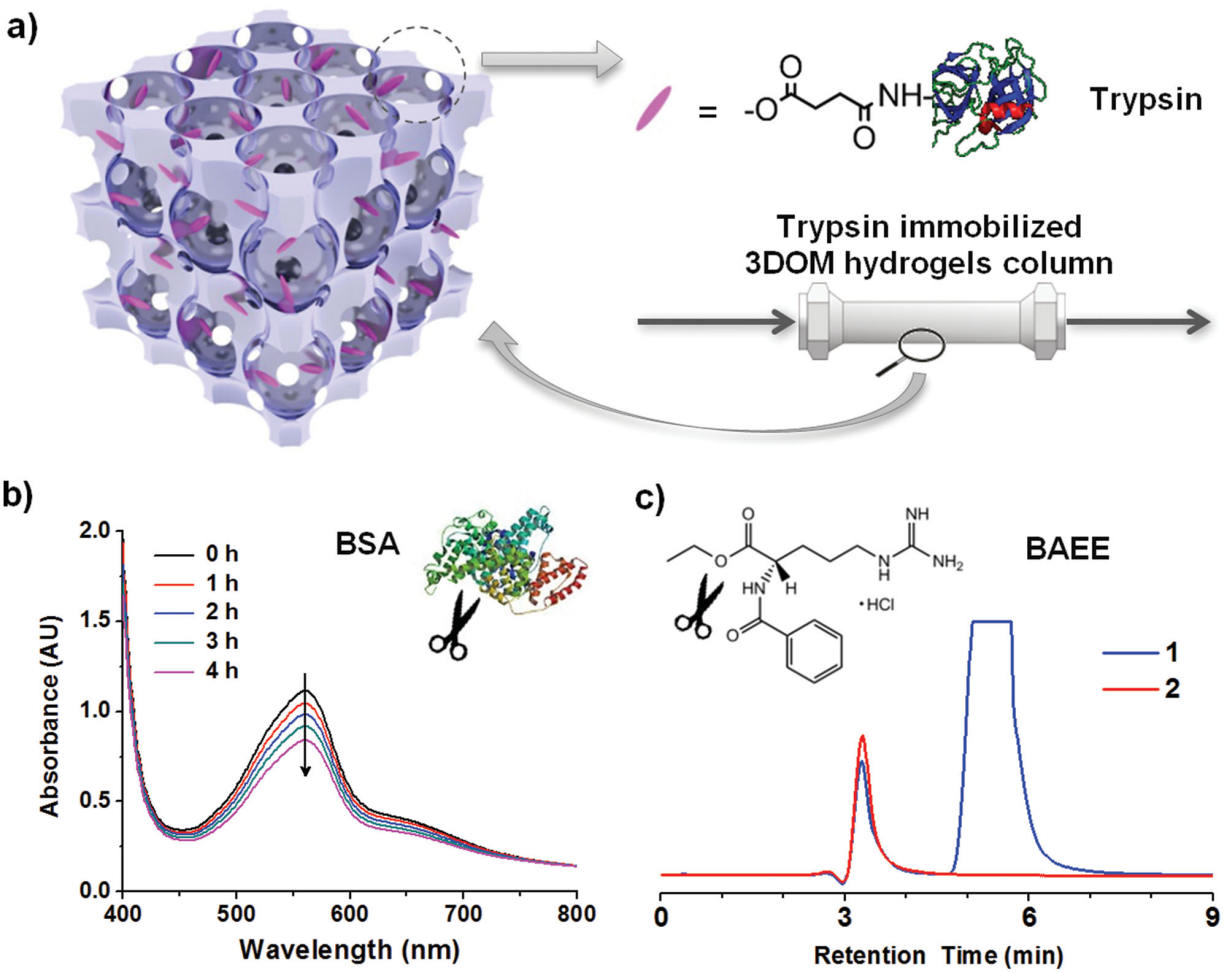
a) Schematic illustration of trypsin immobilized 3DOM hydrogels column. b) The digestion of BSA (0.5 mg mL^−1^, 1 mL) by 3DOM hydrogel‐trypsin (2.5 mg): UV–vis spectra of 25 μL of the BSA solution in 200 μL of bicinchoninic acid (BCA) protein assay reagent (bicinchoninic acid) from 0 to 4 h. The decrease of the absorbance at 562 nm originating from BCA/copper complex indicated the decrease of protein concentrations. c) Online monitoring of the absorption at 223 nm versus retention time in RP18 column for (1) 5 × 10^−3^ m BAEE solution and (2) collected solutions from step 1 with 5 × 10^−3^ m BAEE solution. The disappearance of the peak at ≈5.5 min indicated the complete digestion of BAEE by trypsin immobilized 3DOM hydrogels column.

The 3DOM hydrogels were also loaded with inorganic particles to form novel organic–inorganic composites. This is illustrated by reduction of AuCl_3_ by NaBH_4_ on the surface of 3DOM hydrogels modified with the carboxylic acid groups to form Au nanoparticles (NPs) with an average diameter of 7.1 nm that were uniformly distributed on the pores. The distribution of the formed AuNPs can be observed in the transmission electron microscopy (TEM) images of a thin‐section of the sample (**Figure** **5**b,c, and Supporting Information, Figure S27). The 3DOM hydrogel‐Au NPs composite displayed high catalyst activity as a heterogeneous catalyst for the reduction of 4‐nitrophenol (Figure 5d and Supporting Information, Figure S28).

**Figure 5:**
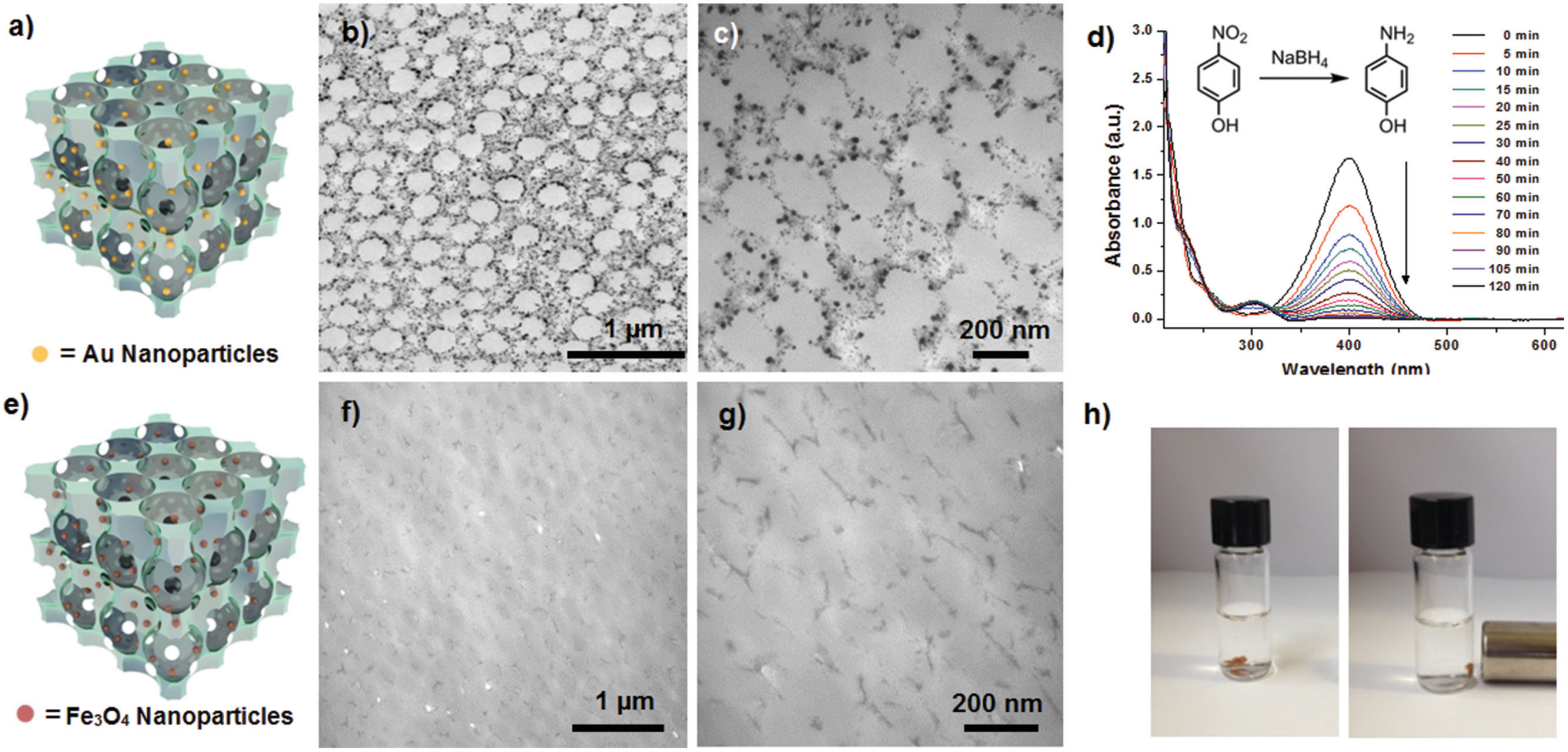
Upper row: 3DOM hydrogels loaded with Au NPs: a) a cartoon representation of the overall structure, b,c) TEM images of a ≈100 nm thin‐section sample, and d) successive UV–vis spectra for the catalytic reduction of 4‐nitrophenol into 4‐aminophenol. Lower row: 3DOM hydrogels loaded with Fe_3_O_4_ NPs: e) a cartoon representation of the overall structure, f,g) TEM images a ≈100 nm thin‐section sample, and h) photos of the sample in water in the absence (left) and presence (right) of a magnetic field.

Another example of the organic–inorganic composite is a magnetic 3DOM hydrogel nanocomposite. By electrostatic self‐assembling, negatively charged magnetite (Fe_3_O_4_) NPs with an average diameter of 3.1 nm were decorated onto the positively charged pore surfaces of 3DOM hydrogels containing quaternary ammonium groups, affording magnetic 3DOM hydrogels (Figure 5e–h and Supporting Information, Figures S29 and S30).

In summary, 3DOM hydrogels were prepared by either conventional FRP or ATRP of hydrophilic functional monomers and crosslinkers in the presence of latex colloidal crystals as the template. This was confirmed by simple and effective visualization of the 3DOM structures through both indirect electron microscopy and direct nano‐XRM characterizations. The highly reversible 3DOM structure of the hydrogels was demonstrated by multiple drying/swelling cycles that indicated the shape‐memory nature of 3DOM hydrogels. Further modifications of functional 3DOM hydrogels with organic moieties, polymers, bio(macro)molecules, and inorganic particles generates materials with new properties (e.g., hydrophobicity, fluorescence, conductivity, and stimuli‐responsivity) into the structured 3DOM hydrogels and result in novel composites for (bio)catalysis and separation applications. The unique 3DOM structures with well‐controlled pore size and excellent pore interconnectivity can also serve as models for systematic studies of the influence of pore size and pore interconnectivity on the properties of the resulting materials (such as fluid mechanics of the flow through pores, catalytic efficiency, etc.). This work suggests that functional 3DOM hydrogels represent a versatile platform for a wide range of applications and there are even more multifunctional materials based on 3DOM gels to be explored.

## Supporting information

As a service to our authors and readers, this journal provides supporting information supplied by the authors. Such materials are peer reviewed and may be re‐organized for online delivery, but are not copy‐edited or typeset. Technical support issues arising from supporting information (other than missing files) should be addressed to the authors.

Supplementary

Supplementary

Supplementary

Supplementary

## References

[1] a) K. Y. Lee , D. J. Mooney , Chem. Rev. 2001, 101, 1869; 11710233 10.1021/cr000108x

[2] a) J. Kopecek , Biomaterials 2007, 28, 5185; 17697712 10.1016/j.biomaterials.2007.07.044 PMC2212614

[3] a) D. Wu , F. Xu , B. Sun , R. Fu , H. He , K. Matyjaszewski , Chem. Rev. 2012, 112, 3959; 22594539 10.1021/cr200440z

[4] a) B. Ye , H. Ding , Y. Cheng , H. Gu , Y. Zhao , Z. Xie , Z. Gu , Adv. Mater. 2014, 26, 3270; 24550084 10.1002/adma.201305035

[5] a) J. Kim , S. A. Bencherif , W. A. Li , D. J. Mooney , Macromol. Rapid Commun. 2014, 35, 1578; 25113941 10.1002/marc.201400278 PMC4318565

[6] a) H. He , W. Li , M. Zhong , D. Konkolewicz , D. Wu , K. Yaccato , T. Rappold , G. Sugar , N. E. David , K. Matyjaszewski , Energy Env. Sci. 2013, 6, 488;

[7] a) Y. N. Xia , B. Gates , Y. D. Yin , Y. Lu , Adv. Mater. 2000, 12, 693;

[8] J. W. Goodwin , J. Hearn , C. C. Ho , R. H. Ottewill , Colloid. Polym. Sci. 1974, 252, 464.

[9] a) K. Matyjaszewski , J. H. Xia , Chem. Rev. 2001, 101, 2921; 11749397 10.1021/cr940534g

[10] H. He , B. Adzima , M. Zhong , S. Averick , R. Koepsel , H. Murata , A. Russell , D. Luebke , A. Takahara , H. Nulwala , K. Matyjaszewski , Polym. Chem. 2014, 5, 2824.

[11] a) S. H. Lau , W. K. S. Chiu , F. Garzon , H. Chang , A. Tkachuk , M. Feser , W. Yun , J. Phys.: Conf. Ser. 2009, 152, 012059;

[12] P. Mandal , S. Litster , ECS Trans. 2013, 58, 481.

[13] Y. Jiang , C. Cui , Y. Huang , X. Zhang , J. Gao , Chem. Commun. 2014, 50, 5490. 10.1039/c4cc01721h 24722982

[14] F. Audouin , M. Fox , R. Larragy , P. Clarke , J. Huang , B. O'Connor , A. Heise , Macromolecules 2012, 45, 6127.

