# Supplementary material for: Multifunctional Hydrogels with Reversible 3D Ordered Macroporous Structures
Source: Adv Sci (Weinh). 2015 Mar 26;2(5):1500069. doi: 10.1002/advs.201500069 (PMC5115371; doi:10.1002/advs.201500069)
Supplement: Supplementary file 1 — Supplementary [file ADVS-2-0h-s002.pdf]

Copyright WILEY-VCH Verlag GmbH & Co. KGaA, 69469 Weinheim, Germany, 2015.

ADVANCED SCIENCE
Open Access

Supporting Information

for *Adv. Sci.*, DOI: 10.1002/advs. 201500069

Multifunctional Hydrogels with Reversible 3D Ordered Macroporous Structures

Hongkun He, Saadyah Averick, Pratiti Mandal, Hangjun Ding, Sipei Li, Jeff Gelb, Naomi Kotwal, Arno Merkle, Shawn Litster, and Krzysztof Matyjaszewski**

Supporting Information:

Multifunctional Hydrogels with Reversible 3D Ordered Macroporous Structures

Hongkun He,[1] Saadyah Averick,[1] Pratiti Mandal,[2] Hangjun Ding,[1,3] Sipei Li,[1] Jeff Gelb,[4] Naomi Kotwal,[4] Arno Merkle,[4] Shawn Litster,[2,*] and Krzysztof Matyjaszewski[1,*]

[1] *Center for Macromolecular Engineering, Department of Chemistry, Carnegie Mellon University, Pittsburgh, Pennsylvania 15213, USA*

[2] *Department of Mechanical Engineering, Carnegie Mellon University, Pittsburgh, Pennsylvania 15213, USA*

[3] *School of Materials Science and Engineering, University of Science & Technology Beijing, 30 Xueyuan Road, Beijing 100083, People's Republic of China*

[4] *Carl Zeiss X-Ray Microscopy, Inc., 4385 Hopyard Road, Pleasanton, California 94588, USA*

*Corresponding author, litster@andrew.cmu.edu, km3b@andrew.cmu.edu.

Experimental section

Materials. Potassium persulfate (KPS, ≥99.0%), potassium sulfate (K_2SO_4, ≥99.0%), α,α'-dichloro-*p*-xylene (98%), *p*-(vinylbenzyl)trimethylammonium chloride (VBTMACl, 99%), 2,2'-bipyridine (≥99%), succinic anhydride (≥99%), triethylamine (≥99%), *N*,*N*-(dimethylamino)pyridine (DMAP, ≥99%), *N*-(3-dimethylaminopropyl)-*N'*-ethylcarbodiimide hydrochloride (EDC•HCl, ≥98.0%), *N*-hydroxysuccinimide (NHS, 98%), 2,2'-azobis(2-methylpropionitrile) (AIBN, 99%), *N*-α-benzoyl-L-arginine *p*-nitroanilide (BAPNA), dodecanoyl chloride (≥97.5%), $NaBH_4$ (99%), 4-nitrophenol (≥99%), ammonium persulfate (APS, $(NH_4)_2S_2O_8$, 98%), (dimethylaminomethyl)phenol, benzyl chloride (99%), rhodamine B (≥95%), 2-aminoethyl methacrylate hydrochloride (90%), α-bromoisobutyryl bromide (98%), ethyl 2-bromoisobutyrate (98%), *N*,*N*,*N'*,*N''*,*N''*-pentamethyldiethylenetriamine (PMDETA, 99%), and poly(acrylic acid) (M_w=1800) were purchased from Aldrich. Styrene (≥99%), methyl methacrylate (MMA, ≥98.5%), 4-vinylbenzyl chloride (CMS, ≥90%), divinylbenzene (DVB, 80%), poly(ethylene oxide) methacrylate (PEOMA, M_n=526), poly(ethylene oxide) dimethacrylate (PEODMA, M_n=750), lauryl methacrylate (96%), and aniline (≥99.5%) were purchased from Aldrich and purified by passing over a column of basic alumina to remove the inhibitor. *N*-Isopropylacrylamide (≥99%, Aldrich) was recrystallized twice from benzene/hexane prior to use. Diethylene glycol (99%), gold(III) chloride ($AuCl_3$, Au 64.4% min), and trypsin (bovine pancreas, minimum 2500 USP units/mg) were purchased from Alfa. Tris[2-(dimethylamino)ethyl]amine (Me_6TREN, 99%) was purchased from ATRP Solutions. NaOH (≥98%) was purchased from Fisher Scientific. *N*-(4-vinylbenzyl)-*N,N*-dimethylamine (90%) was purchased from Acros Organics. 2,2'-Azobis(2-amidinopropane) dihydrochloride (V50) and 2,2'-azobis[2-(2-imidazolin-2-yl)propane]dihydrochloride (VA-044) were purchased from Wako Pure Chemical Industries Ltd. CuCl, $CuCl_2$, CuBr, and $CuBr_2$ were purchased from Aldrich in the highest available purity. Unless otherwise specified all reagents were used as received. *N*,*N'*-(1,4-phenylenebis(methylene))bis(*N*,*N*-dimethyl-1-(4-vinylphenyl)methanamonium) dichloride (PMVPMACl) was synthesized following previously published procedures.[1] Poly(ethylene oxide) isobutyryl bromide (PEO_{2000}iBBr, M_n = 2,000) was synthesized following previously published procedures.[2] PMMA colloidal crystals were prepared from monodisperse colloidal PMMA spheres, which were synthesized by surfactant-free emulsion polymerization using an optimized version of literature techniques,[3-4] as described elsewhere.[1]

Instrumentation. Proton nuclear magnetic resonance (^{1}H NMR) measurements were performed on a Bruker Avance 300 MHz spectrometer. Elemental analysis was determined at Midwest Microlab, LLC. Scanning electron microscopy (SEM) analysis was conducted using a Hitachi 2460N scanning electron microscope. The specimens were attached to SEM stubs using rubber cement or double sided sticky tape, and coated with gold using a Pelco SC-6 sputter coater. Transmission electron microscopy (TEM) analysis was conducted using a Hitachi H-7100 transmission electron microscope (Hitachi High Technologies America) operating at 75 kV. For the ultra-thin sections TEM measurements, the samples were infiltrated with 100% Epon-Araldite or LR White for several hours, and placed in embedding capsules. The capsules were polymerized at 60 ^{o}C for 24 h. Thin (100 nm) sections were cut using a DDK diamond knife on a Reichert-Jung Ultracut E ultramicrotome. Molecular weight and molecular weight distribution (M_w/M_n) were determined by gel permeation chromatography (GPC). The GPC system used a Waters 515 HPLC pump and a Waters 2414 refractive index detector using PSS columns (Styrogel 10^2, 10^3, 10^5 Å) with tetrahydrofuran (THF) as the eluent at a flow rate of 1 mL/min at 35 °C, or dimethylformamide (DMF) containing 50 mM LiBr as the eluent at a flow rate of 1 mL/min at 50 °C. Nano-scale resolution X-ray microscopy (nano-XRM) or nano-scale resolution computed tomography (nano-CT) was performed using an Xradia (now Carl Zeiss X-ray Microscopy, Pleasanton, CA) UltraXRM-L200. The image visualization and segmentation were performed using Avizo® Fire 8.1 (Visualization Sciences Group, Burlington, MA).

Preparation of 3DOM hydrogels by colloidal crystal templating via conventional free radical copolymerization (FRP). Typically, 0.5 g of PMMA colloidal crystals were placed in a 20 mL glass vial which was then sealed with a rubber stopper. The vial was put in vacuum for 5 min and then purged with nitrogen. The vacuum/purge cycle was repeated five times. The aqueous solution of monomer/crosslinker/initiator mixture was degased and then added via to the vial gas-tight syringe. The polymerization was carried out at 50 °C. The surface layer of the resulting composite without incorporated colloidal crystal templates was removed by cutting with a razor blade. The PMMA template was removed from the sample by extraction with acetone over one day.

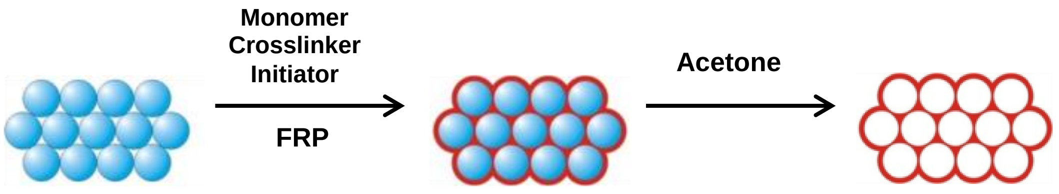

Preparation of 3DOM hydrogels by colloidal crystal templating via atom transfer radical polymerization (ATRP). Typically, 0.5 g of PMMA colloidal crystals were placed in a 20 mL glass vial which was then sealed with a rubber stopper. The vial was put under vacuum for 5 min and then purged with nitrogen. The vacuum/purge cycle was repeated five times. The degased aqueous solution of the catalyst (CuCl/$CuCl_2$/bipyridine), monomer, and crosslinker was added to the vial via gas-tight syringe. The polymerization was carried out at 25 °C. The surface layer of the resulting composite without incorporated colloidal crystal templates was removed by cutting with razor blade. The PMMA template was removed from the sample by extraction with acetone over one day.

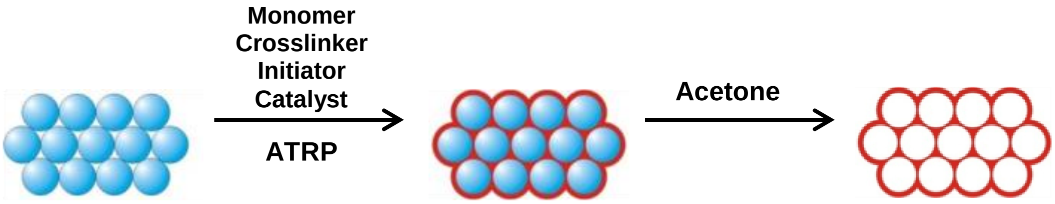

Synthesis of *N*-benzyl-1-(4-(methacryloyloxy)phenyl)-*N,N*-dimethylmethanaminium chloride (BMDAC). (Dimethylaminomethyl)phenol (3.6 g, 23.7 mmol) was added to methanol (6.6 mL) followed by sequential addition of benzyl chloride (3.0 g, 23.7 mmol) and deionized water (2.1 mL). The mixture was heated at 70 ^{o}C for 24 h. After cooling to room temperature, the reaction solution was concentrated and precipitated into cold acetone. The precipitate was dried in vacuum at 50 ^{o}C to give *N*-benzyl-1-(4-hydroxyphenyl)-*N,N*-dimethylmethanaminium chloride. ^{1}H NMR (D_2O, δ, ppm): 2.9 (s, 6H), 4.5 (s, 4H), 6.8-7.7 (m, 9H). A mixture of *N*-benzyl-1-(4-hydroxyphenyl)-*N,N*-dimethylmethanaminium chloride (10.75 g, 38.7 mmol), methacrylic acid (5.00 g, 58.0 mmol), DCC (11.98 g, 58.0 mmol), and DMAP (0.71 g, 5.8 mmol) in anhydrous DMF (45 mL) was stirred at room temperature for 18 h. The reaction mixture was filtered, precipitated in acetone, and dried in vacuum at room temperature to give *N*-benzyl-1-(4-(methacryloyloxy)phenyl)-*N,N*-dimethylmethanaminium chloride. ^{1}H NMR (D_2O, δ, ppm): 1.9 (s, 3H), 2.9 (s, 6H), 4.5 (d, 4H), 5.8 (d, 1H), 6.2 (d, 1H), 6.8-8.0 (m, 9H).

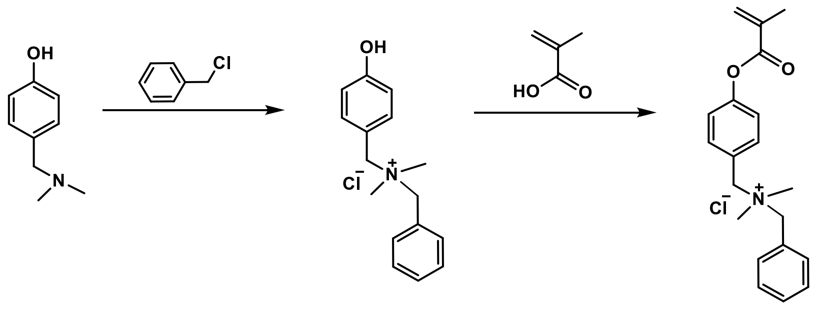

Preparation of 3DOM hydrogels containing -OH groups (PEODMA/PEOMA-OH, with degree of crosslinking 20%): The procedure was the same as that used for the preparation of 3DOM hydrogel by colloidal crystal templating via conventional FRP with the following ratio of reagents. Poly(ethylene oxide) dimethacrylate (PEODMA, M_n=750, 1.00 g, 1.3 mmol), poly(ethylene oxide) methacrylate (PEOMA, M_n=526, 2.81 g, 5.3 mmol), 2,2'-azobis(2-methylpropionamidine dihydrochloride (VA-044, 1.8 mg) and H_2O (3.81 g).

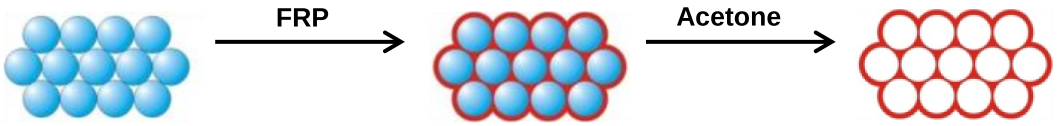

Preparation of 3DOM hydrogels containing -COOH groups (PEODMA/PEOMA-COOH): Succinic anhydride (1.40 g, 14 mmol), triethylamine (0.14 g, 1.4 mmol), and *N*,*N*-(dimethylamino)pyridine (DMAP, 17 mg, 0.14 mmol) were dissolved in 4 mL of anhydrous acetone. A sample of 3DOM hydrogel PEODMA/PEOMA-OH (1.0 g, *ca.* 1.4 mmol –OH group) was taken out of acetone and added into the solution. The mixture was shaken for 1 day at 60 ^{o}C and was then washed with excess acetone. Elemental analysis results for the sample prepared from 3DOM PEODMA/PEOMA-OH hydrogel (degree of crosslinking 20%) showed that the content of C changed from 53.61% to 53.13% after the –OH groups were converted to –COOH groups, which corresponded to 0.94 mmol –COOH/g.

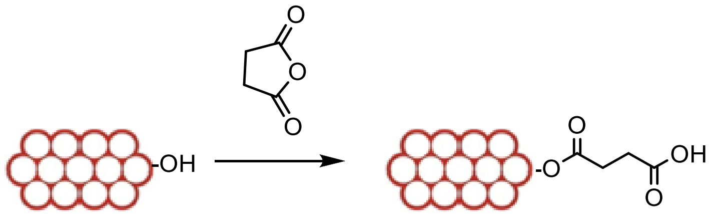

Preparation of 3DOM hydrogels containing trypsin (PEODMA/PEOMA-Trypsin): Trypsin (0.3 g, minimum 2500 USP units/mg), *N*-(3-Dimethylaminopropyl)-*N'*-ethylcarbodiimide hydrochloride (EDC•HCl, 0.28 g, 1.5 mmol), and *N*-hydroxysuccinimide (0.17 g, 0.15 mmol) were dissolved into 4 mL TRIS (PH=8.0) solution. A sample of the 3DOM PEODMA/PEOMA-COOH hydrogel (1.0 g, *ca.* 1.5 mmol –COOH group) was taken out of acetone and added into the solution. The mixture was shaken for 1 day at room temperature. The product was washed with TRIS (PH=8.0).

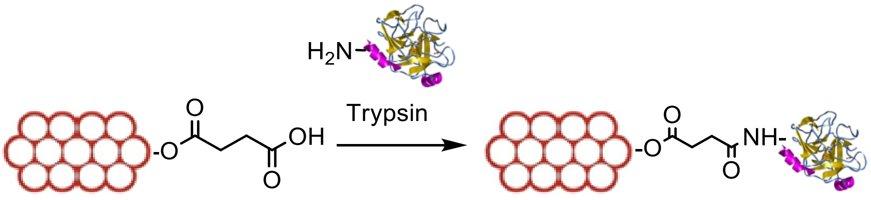

Preparation of 3DOM hydrogels containing long alkyl chains (PEODMA/PEOMA-C12): Dodecanoyl chloride (0.153 g, 0.7 mmol), triethylamine (8.1 mg, 0.07 mmol), and DMAP (8.6 mg, 0.07 mmol) were dissolved in 2 mL anhydrous THF. Then, 3DOM PEODMA/PEOMA-COOH hydrogel (5 mg) was added to the solution. The mixture was shaken for 1 day at room temperature. The product was washed with THF.

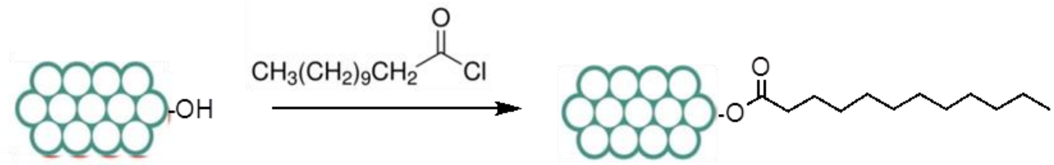

Preparation of 3DOM hydrogels containing Au nanoparticles (PEODMA/PEOMA-Au): 3DOM PEODMA/PEOMA-COOH hydrogel (5 mg) was added to 2 mL of an aqueous solution of $AuCl_3$ (21 mg, 0.07 mmol). Then, 2.1 mL of $NaBH_4$ aqueous solution (0.1 M) was added dropwise to the mixture. The mixture was shaken for 1 day at room temperature. The product was washed with water.

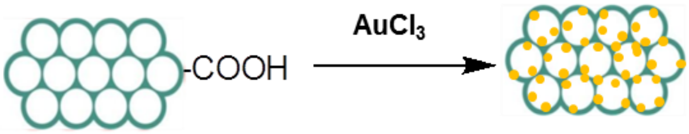

Measurements of the catalytic properties of the 3DOM PEODMA/PEOMA-Au hydrogel: Deionized water (1.0 mL), 4-nitrophenol aqueous solution (1 mM, 0.1 mL), and $NaBH_4$ aqueous solution (36 mM, 2.0 mL) were added to a standard quartz cuvette with a 1 cm path length,. All solutions were previously deaerated and saturated with N_2. After 3DOM PEODMA/PEOMA-Au hydrogel (2.5 mg) was added to the solution, the absorption spectra were recorded every 30 s at room temperature.

Preparation of 3DOM hydrogels containing polyaniline: Polyaniline was synthesized according to a previously reported procedure.[5] 3DOM hydrogel PEODMA/PEOMA-COOH (5 mg) was soaked in a shaken mixture of aniline and water ([aniline] = 0.5 M) for 1 h. Fresh prepared aqueous solution of APS (36 mM, [aniline]/[ammonium persulfate] = 1/8) was added. After 10 min, the product was washed with water.

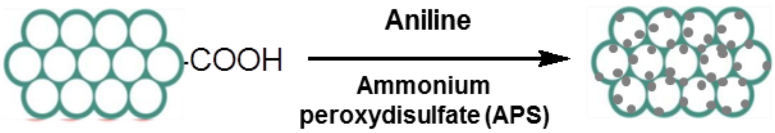

Preparation of Rhodamine B modified 3DOM hydrogels (PEODMA-PEOMA-RhB): 3DOM hydrogel PEODMA/PEOMA-OH (5 mg, *ca.* 7.0 μmol –OH group) was mixed with rhodamine B (13 mg, 28 μmol), EDC HCl (5 mg, 28 μmol), and *N*-hydroxysuccinimide (3 mg, 28 μmol) in 1 mL of water. The mixture was shaken for 1 day at r.t.. The product was removed from the reaction solution and washed with excess water.

Preparation of 3DOM hydrogels containing -NH_4^+ groups (PEODMA/AEMA-NH_4^+ with a degree of crosslinking 20%): The procedure was the same as that used for the preparation of 3DOM hydrogel by colloidal crystal templating via conventional FRP with the following ratio of reagents. Poly(ethylene oxide) dimethacrylate (PEODMA, M_n=750, 1.00 g, 1.3 mmol), 2-aminoethyl methacrylate hydrochloride (AEMA, 0.88 g, 5.3 mmol), 2,2'-azobis(2-methylpropionamidine dihydrochloride (1.8 mg, VA-044), and H_2O (1.88 g).

Preparation of Fe_3O_4 nanoparticles: Fe_3O_4 nanoparticles were synthesized according to a previously reported procedure.[6] NaOH (2.00 g) was added into diethylene glycol (DEG, 20 mL), heated at 120 ℃

for 1 h in a nitrogen atmosphere, and cooled down to 70 ℃ to produce a NaOH/DEG stock solution. In a separate flask, $FeCl_3$ (0.65 g, 4 mmol) and poly(acrylic acid) (M_w=1800, 0.58 g, 8 mmol) were added in DEG (30 mL), and the mixture was heated to 220 ℃ for 30 min under the protection of nitrogen flow and constant stirring. A 70 ℃ NaOH/DEG stock solution (8.0 mL) was injected rapidly into the hot mixture. The resulting mixture was further heated at 220 ℃ for an additional 10 min. The final product was separated by centrifugation and washed with methanol.

Preparation of Fe_3O_4 nanoparticles functionalized 3DOM hydrogels (PEODMA/AEMA-Fe_3O_4): 3DOM PEODMA/AEMA-NH_4^+ hydrogel (5 mg) was added to 5 mL of a suspension of the Fe_3O_4 nanoparticles in methanol (10 mg/mL). The mixture was shaken for 1 day at r.t.. The product was removed from the reaction solution and washed with excess methanol.

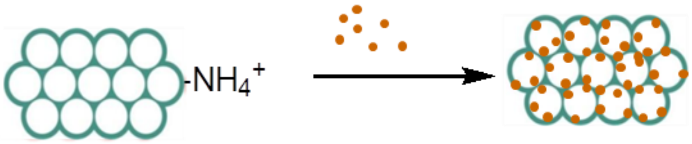

Preparation of ATRP initiator modified 3DOM hydrogels (PEODMA-PEOMA-Br): 3DOM PEODMA/PEOMA-OH hydrogel (5 mg, *ca.* 7.0 μmol –OH group) was mixed with α-bromoisobutyryl bromide (0.16 g, 0.7 mmol) and triethylamine (80 mg, 0.7 mmol) in 1 mL of anhydrous THF. The mixture was shaken for 1 day at r.t.. The 3DOM hydrogel PEODMA-PEO-Br was removed from the reaction solution and washed with excess THF. Elemental analysis result showed that the Br content was 0.98 mmol/g.

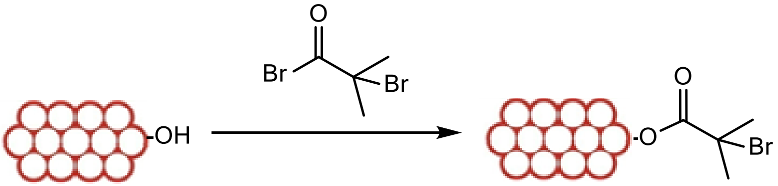

Preparation of polymer grafted 3DOM hydrogels: A sample of 3DOM hydrogel PEODMA-PEOMA-Br, monomer, $CuBr_2$ (or $CuCl_2$), ligand (PMDETA or Me_6TREN), ethyl 2-bromoisobutyrate (EBiB), and solvent were added into a Schlenk flask. The flask was then degassed by three freeze-pump-thaw cycles. While the contents were frozen in liquid nitrogen, the flask was back filled with nitrogen and CuBr (or CuCl) was added. The flask was then degassed and back filled with nitrogen thrice. The

flask was allowed to warm up to room temperature and an initial sample (t = 0) was collected by syringe. The flask was then placed in an oil bath thermostated at the desired temperature. At timed intervals, samples of the reaction mixtures were taken for ^{1}H NMR and GPC measurements. The polymerization was stopped by opening the flask and exposing the catalyst complex in the solution to air. Poly(*N*-isopropylacrylamide) (PNIPAM) was synthesized according to a previously reported procedure.[7] Poly(lauryl methacrylate) was synthesized according to a previously reported procedure.[8-9]

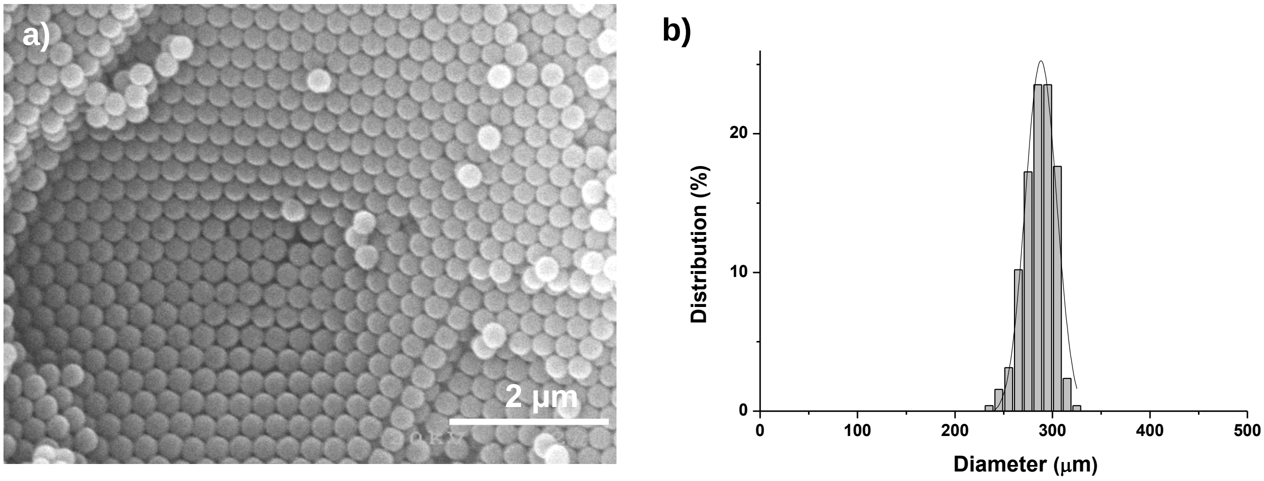

Figure S1. (a) SEM image and (b) the particle size histogram with a Gaussian size distribution fit (solid line) for the PMMA colloidal crystal spheres. 286 ± 15 nm

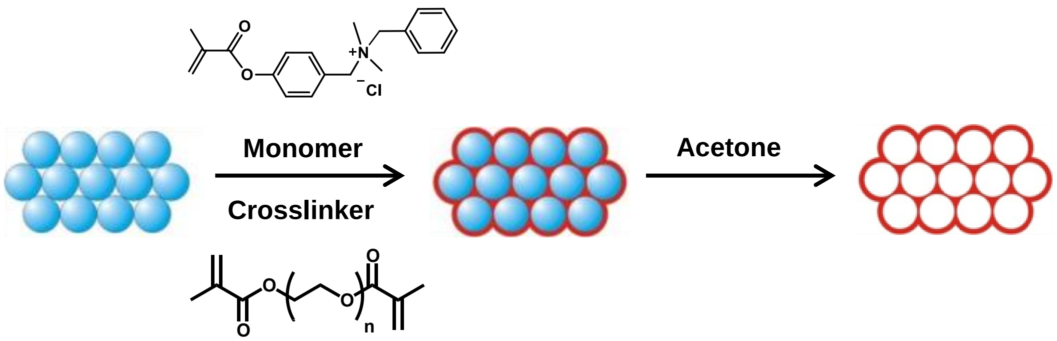

Scheme S1. Preparation of 3DOM hydrogels by colloidal crystal templating via FRP of PEODMA and *N*-benzyl-1-(4-(methacryloyloxy)phenyl)-*N,N*-dimethylmethanaminium chloride (BMDAC).

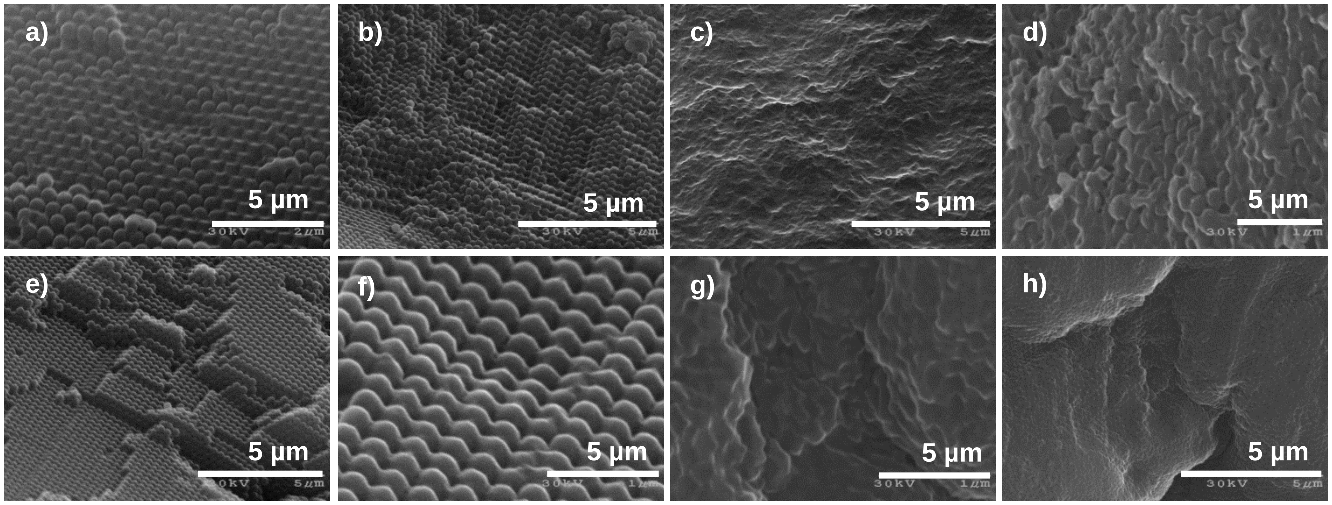

Figure S2. SEM image of 3DOM hydrogels with crosslinking degree of 50% (a-d) and 90% (e-h) before (a,b,e,f) and after (c,d,g,h) removal of the PMMA colloidal crystal templates by washing with acetone. Reaction conditions: (a-d) [BMDAC]/[$PEODMA_{750}$]/[VA-044] = 500/500/1, water/monomer=1/1 (w/w), 50 ^{o}C; (e-h) [BMDAC]/[$PEODMA_{750}$]/[VA-044] = 900/100/1, water/monomer=1/1 (w/w), 50 ^{o}C.

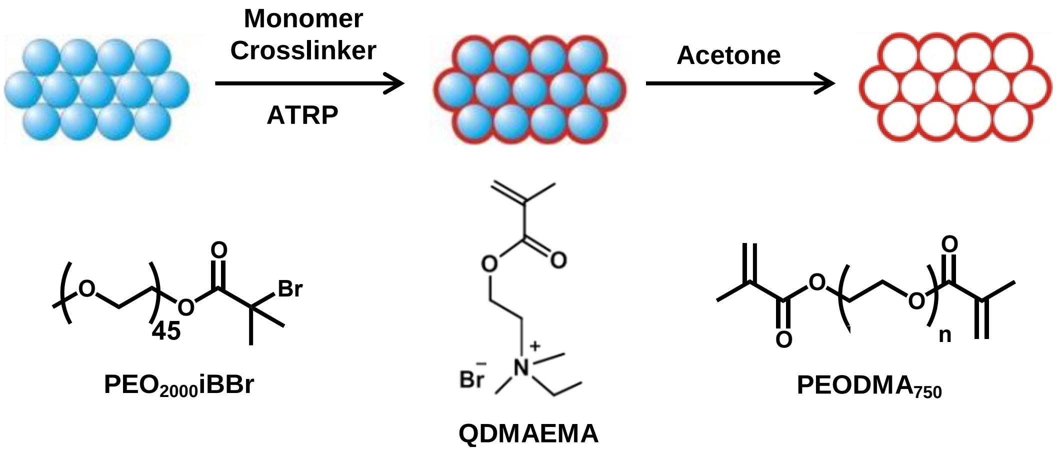

Scheme 2. Preparation of 3DOM hydrogels by colloidal crystal templating via aqueous ATRP of quaternized 2-(dimethylamino)ethyl methacrylate (QDMAEMA).

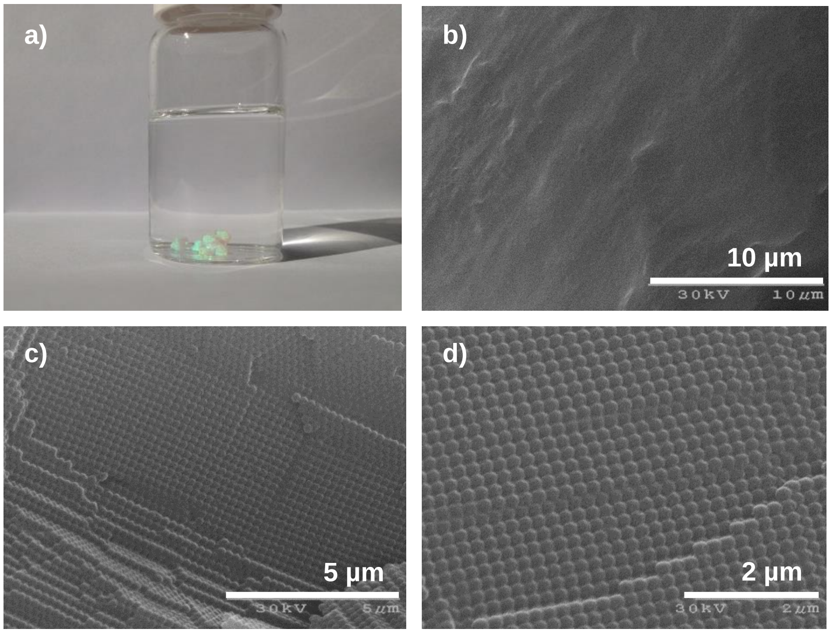

Figure S3. 3DOM hydrogels prepared by aqueous ATRP of quaternized 2-(dimethylamino)ethyl methacrylate (QDMAEMA). (a) Digital photograph of 3DOM hydrogels in acetone under sunlight. (b) SEM image of 3DOM hydrogels after removal of the PMMA colloidal crystal templates by washing with acetone and drying. (c,d) SEM images of 3DOM hydrogels after washing with acetone and loading with DVB/acetone, which were thermally polymerized in situ in the porous structure. Reaction conditions: [PEG_{2000}iBBr]/[QDMAEMA]/[$PEGDMA_{750}$]/[CuCl]/[$CuCl_2$]/[bpy] = 1/120/120/1/9/21, monomer/water = 1/2 (w/w), 25 °C, 5 h.

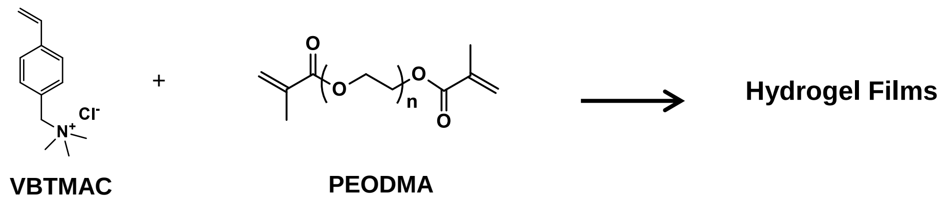

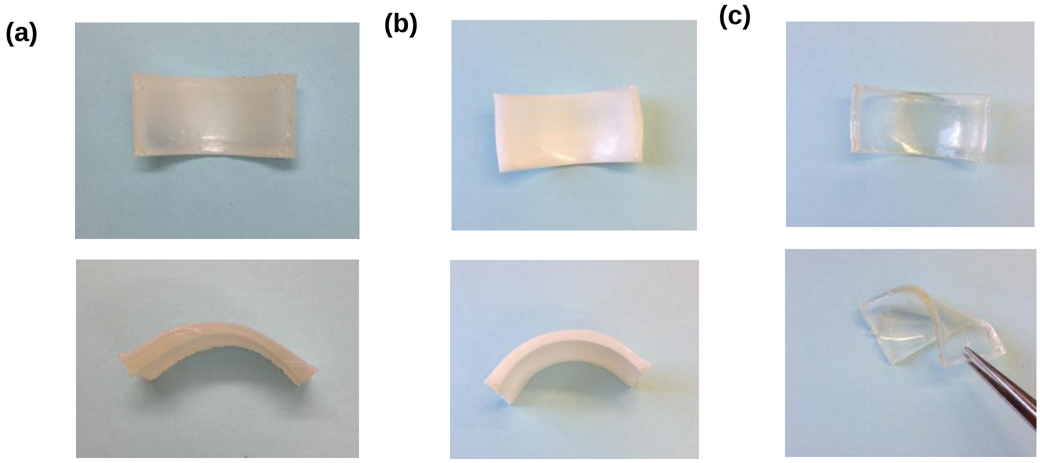

Figure S4. Photos of hydrogel films with different degrees of crosslinking: 10% (a), 50% (b), and 100% (c), respectively. Reaction conditions: (a) [VBTMAC]/[PEODMA]/[VA-044] = 900/100/1, water/VBTMAC =1/1 (w/w), 50 ^{o}C; (b) [VBTMAC]/[PEODMA]/[VA-044] = 500/500/1, water/VBTMAC =1/1 (w/w), 50 ^{o}C; (c) [PEODMA]/[VA-044] = 1000/1, water/PEODMA =1/1 (w/w), 50 ^{o}C.

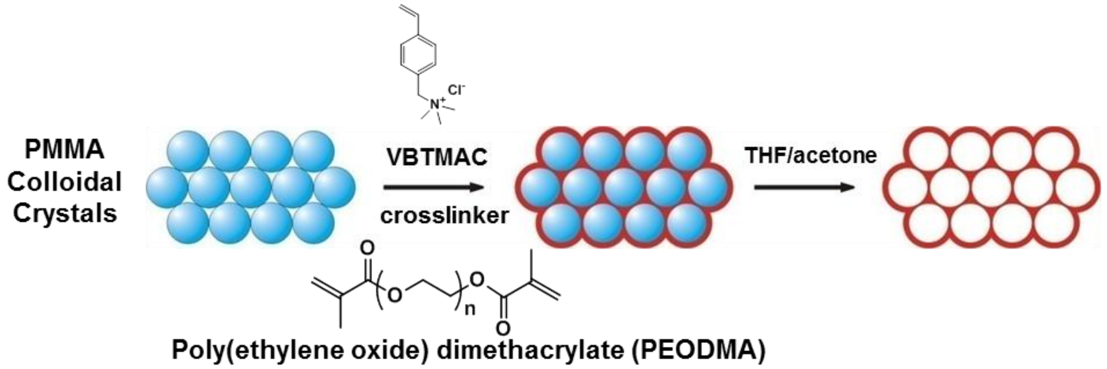

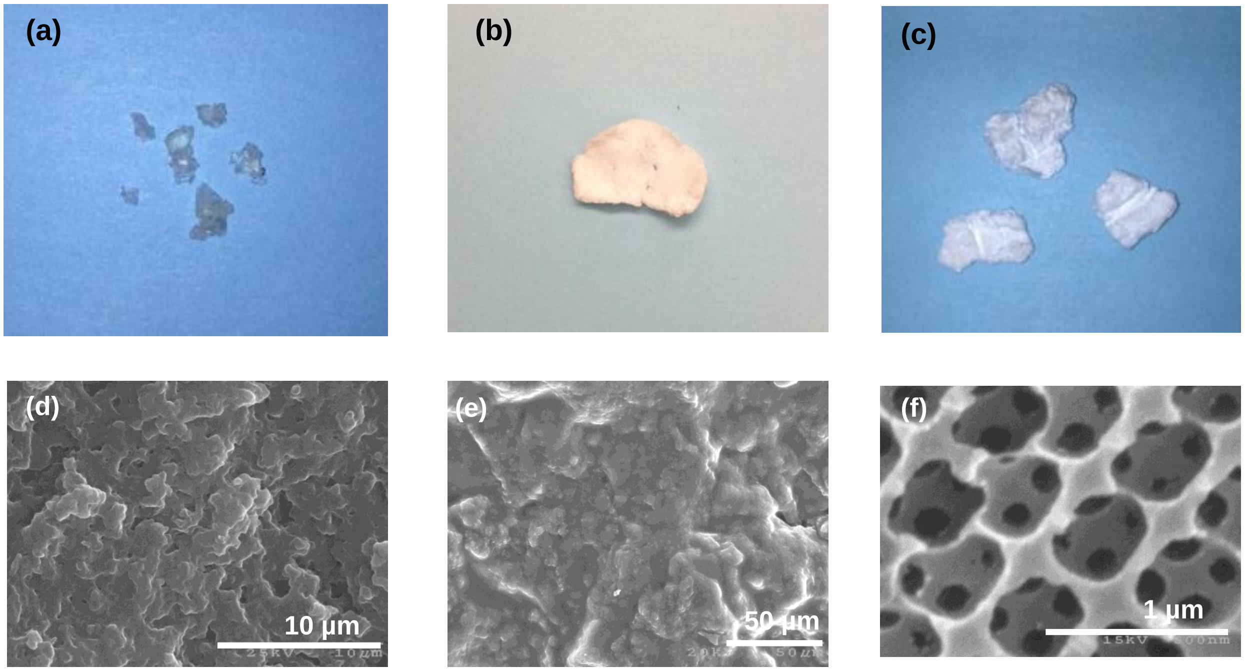

Figure S5. Photos (a-c) and SEM images (d-f) of the 3DOM hydrogels prepared with PMMA colloidal crystal templating by FRP of VBTMAC/PEODMA with different degrees of crosslinking after washing with acetone. Degrees of crosslinking: 100% (a,d), 50% (b,e), and 10% (c,f), respectively. Reaction conditions: (a,d) [PEODMA]/[VA-044] = 1000/1, water/PEODMA =1/1 (w/w), 50 ^{o}C; (b,e) [VBTMAC]/[PEODMA]/[VA-044] = 500/500/1, water/VBTMAC =1/1 (w/w), 50 ^{o}C; (c,f) [VBTMAC]/[PEODMA]/[VA-044] = 900/100/1, water/VBTMAC =1/1 (w/w), 50 ^{o}C.

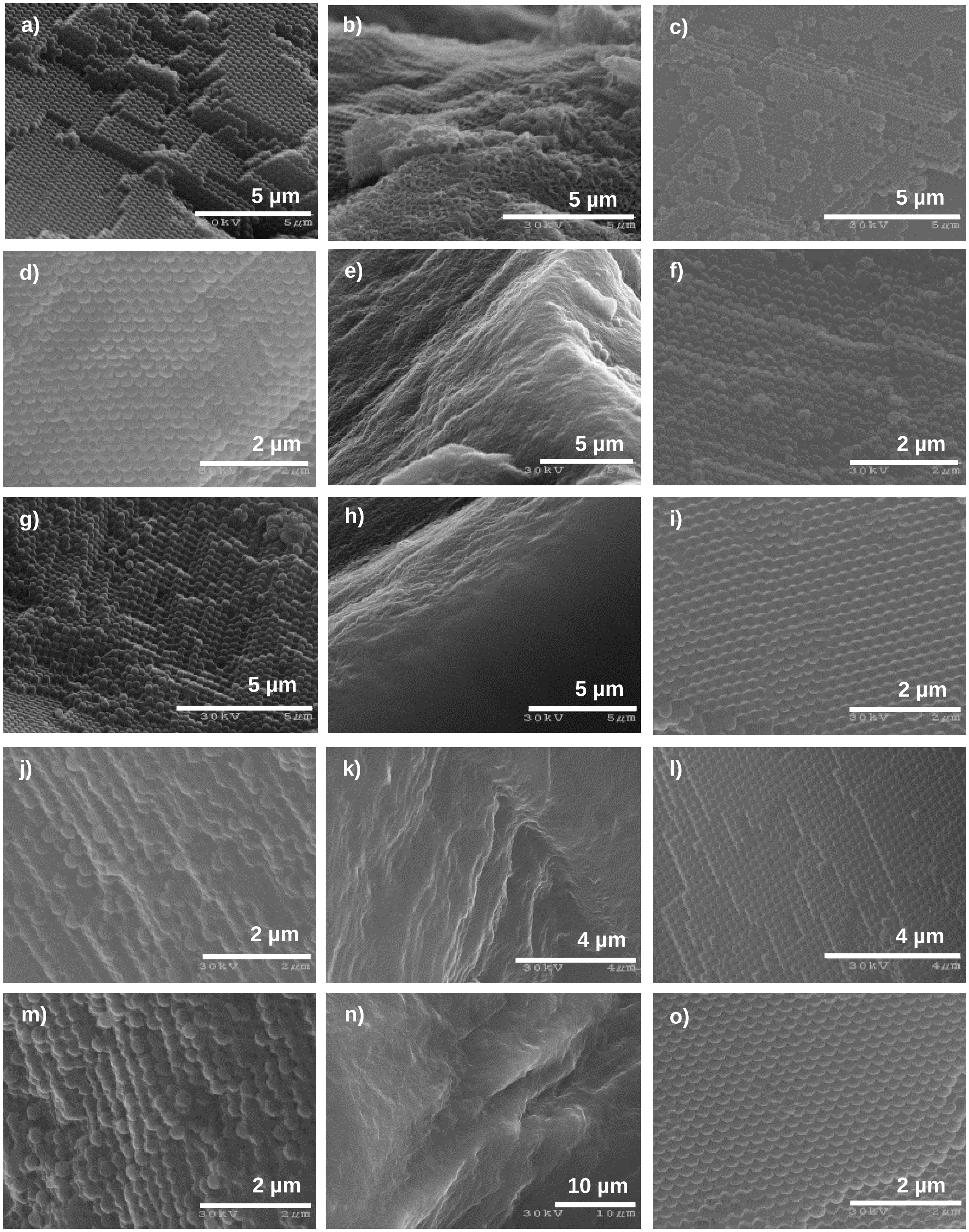
a)
5 µm
b)
5 µm
c)
5 µm
d)
2 µm
e)
5 µm
f)
2 µm
g)
5 µm
h)
5 µm
i)
2 µm
j)
2 µm
k)
4 µm
l)
4 µm
m)
2 µm
n)
10 µm
o)
2 µm

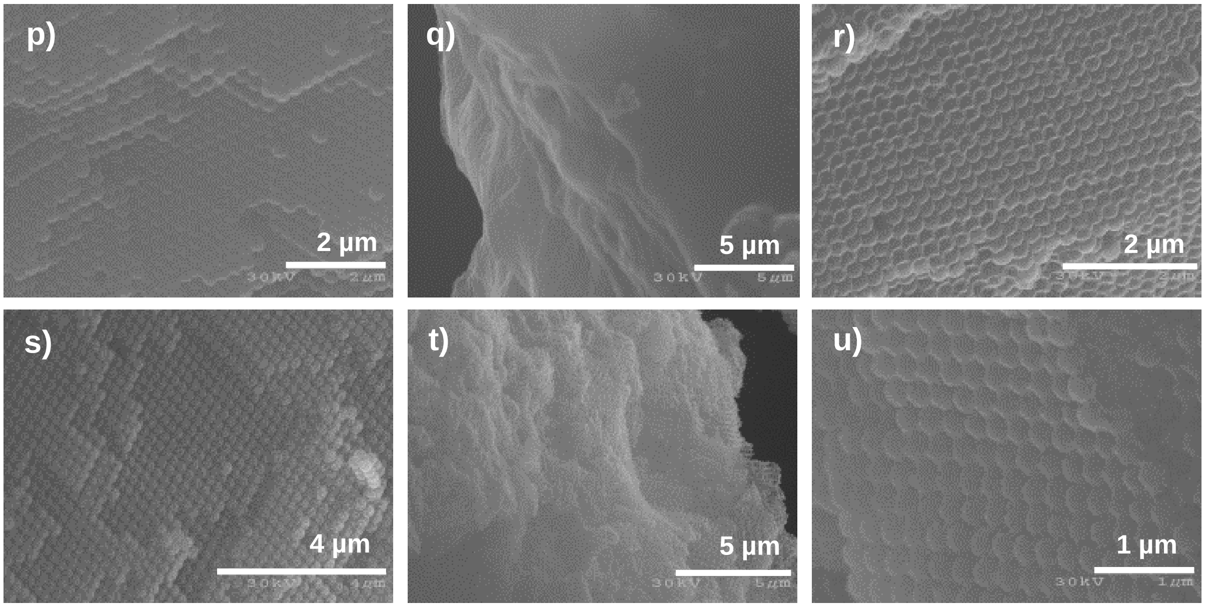

Figure S6. SEM images of the 3DOM hydrogels prepared with PMMA colloidal crystal templating by FRP of PEODMA-QDMAEMA with different degrees of crosslinking: 90% (a-c), 70% (d-f), 50% (g-i), 30% (j-l), 10% (m-o), 0% (p-r), and 100% (s-u). Before washing with acetone: a, d, g, j, m, p, and s. After washing with acetone: b, e, h, k, n, q, and t. After 10 cycles of drying/re-swelling and loaded with DVB then FRP of DVB in situ in the pores, SEM images of the section surfaces of the resulting materials: c, f, i, l, o, r, and u. Reaction conditions: [monomer+crosslinker]/[VA-044] = 1000/1, water/(monomer+ crosslinker) = 1/1 (w/w), 50 ^{o}C.

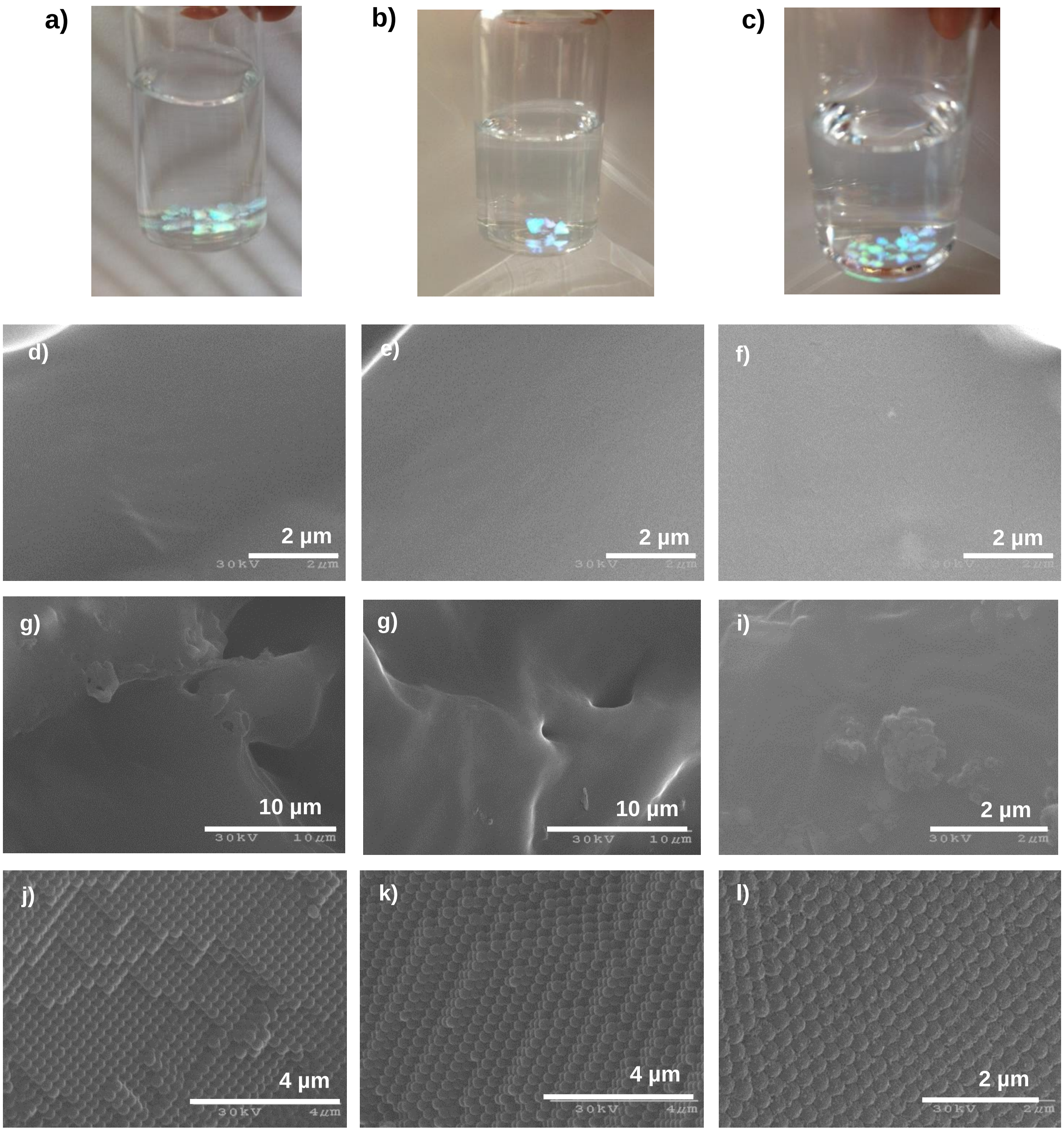

Figure S7. Characterizations of the 3DOM hydrogels prepared by colloidal crystal templating via aqueous ATRP of $OEOMA_{300}$ and $PEOMA_{750}$ with $PEO_{2000}iBBr$ as the initiator. (a-c) Digital

photographs of the 3DOM hydrogels under sunlight after removal of the PMMA colloidal crystal templates by washing with acetone) in acetone. (d-i) SEM images of the 3DOM hydrogels after removal of the PMMA colloidal crystal templates by washing with acetone. (j-l) SEM images of the 3DOM hydrogels after removal of the PMMA colloidal crystal templates by washing with acetone, and loading with DVB/acetone, which were thermally polymerized in situ in the porous structures. Reaction conditions: (a,d,g,j) [PEG_{2000}iBBr]/[$OEOMA_{300}$]/[$PEGDMA_{750}$]/[CuCl]/[$CuCl_2$]/[bpy] = 1/120/8/1/9/21, monomer/water = 1/2 (w/w), 25 °C, 5 h. (a,d,g,j) [PEO_{2000}iBBr]/[$OEOMA_{300}$]/[$PEGDMA_{750}$]/[CuCl]/[$CuCl_2$]/[bpy] = 1/120/25/1/9/21, monomer/water = 1/2 (w/w), 25 °C, 5 h. (a,d,g,j) [PEO_{2000}iBBr]/[$OEOMA_{300}$]/[$PEGDMA_{750}$]/[CuCl]/[$CuCl_2$]/[bpy] = 1/120/45/1/9/21, monomer/water = 1/2 (w/w), 25 °C, 5 h.

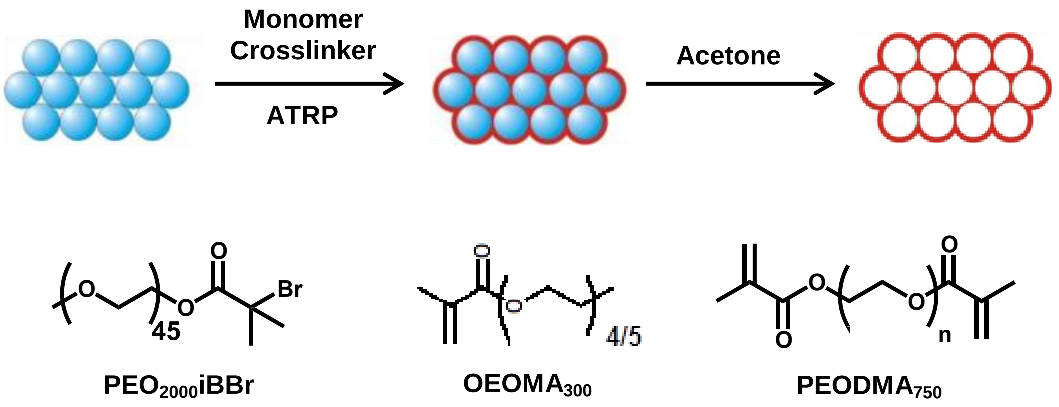

Scheme 3. Preparation of 3DOM hydrogels by colloidal crystal templating via aqueous ATRP of oligo(ethylene oxide) methyl ether methacrylate ($OEOMA_{300}$, M_n = 300) and poly(ethylene oxide) dimethacrylate ($PEOMA_{750}$, M_n = 750) with poly(ethylene oxide) isobutyryl bromide (PEO_{2000}iBBr, M_n = 2,000) as the initiator.

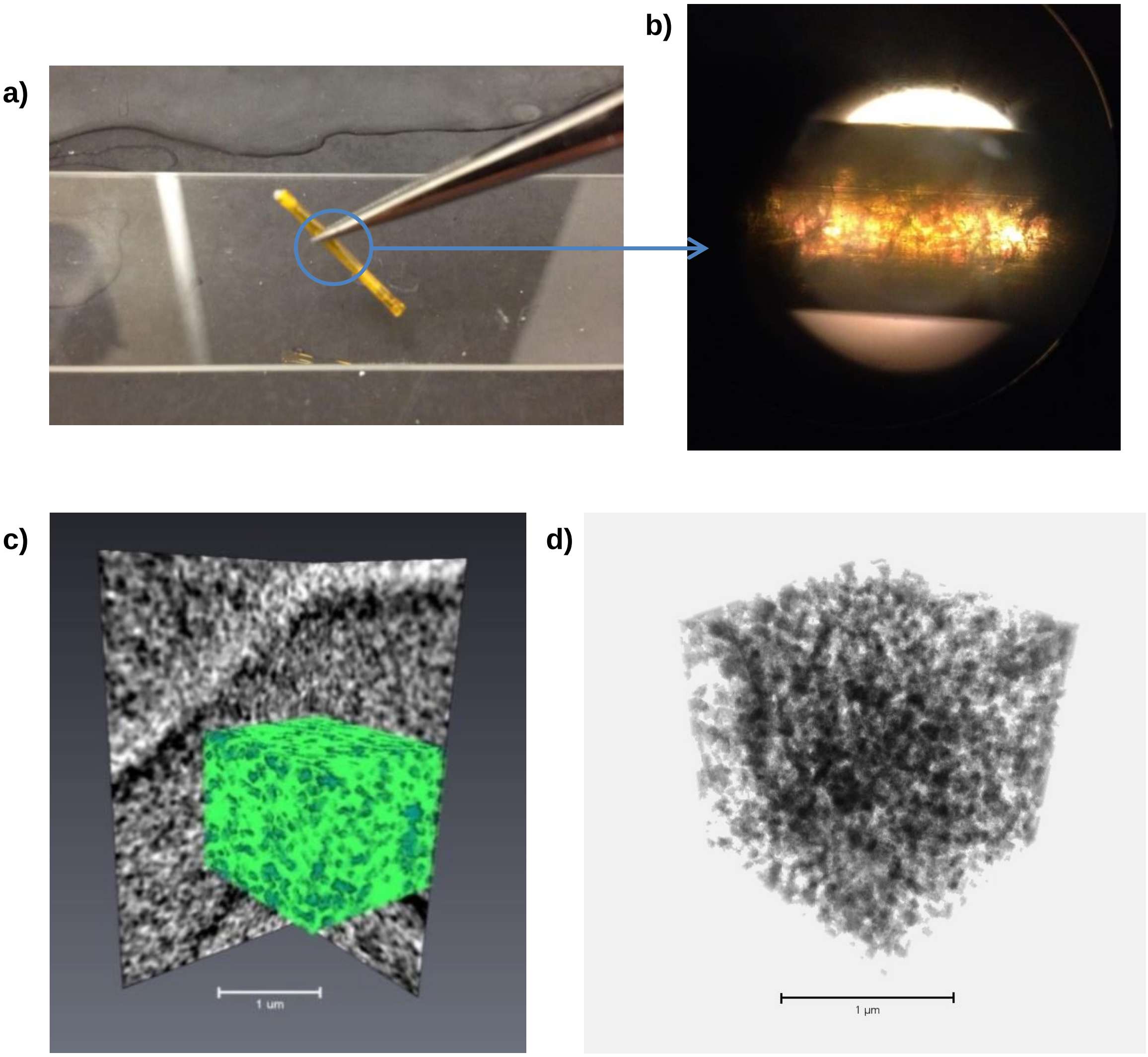

Figure S8. (a) A Kapton tube (inner diameter: 1 mm) loaded with 3DOM hydrogels filled with water and sealed for imaging using nano-XRM. Gold particles with diameter 0.8-1.5 μm diameter were sprinkled on the hydrogel pieces before loading them into the tube to track and correct for sample drift during imaging. (b) Visible light microscope image of 3DOM hydrogels. (c) Additional 3D nano-XRM images of the trypsin immobilized 3DOM hydrogels soaked in water. The orthogonal tomography slices through the reconstructed volume in grayscale is shown along with a volume rendering of a cropped part reconstructed volume after image segmentation. Dark phase corresponds to pores. (d) Volume rendering of the pore phase in the cropped volume of reconstructed image with increased transparency.

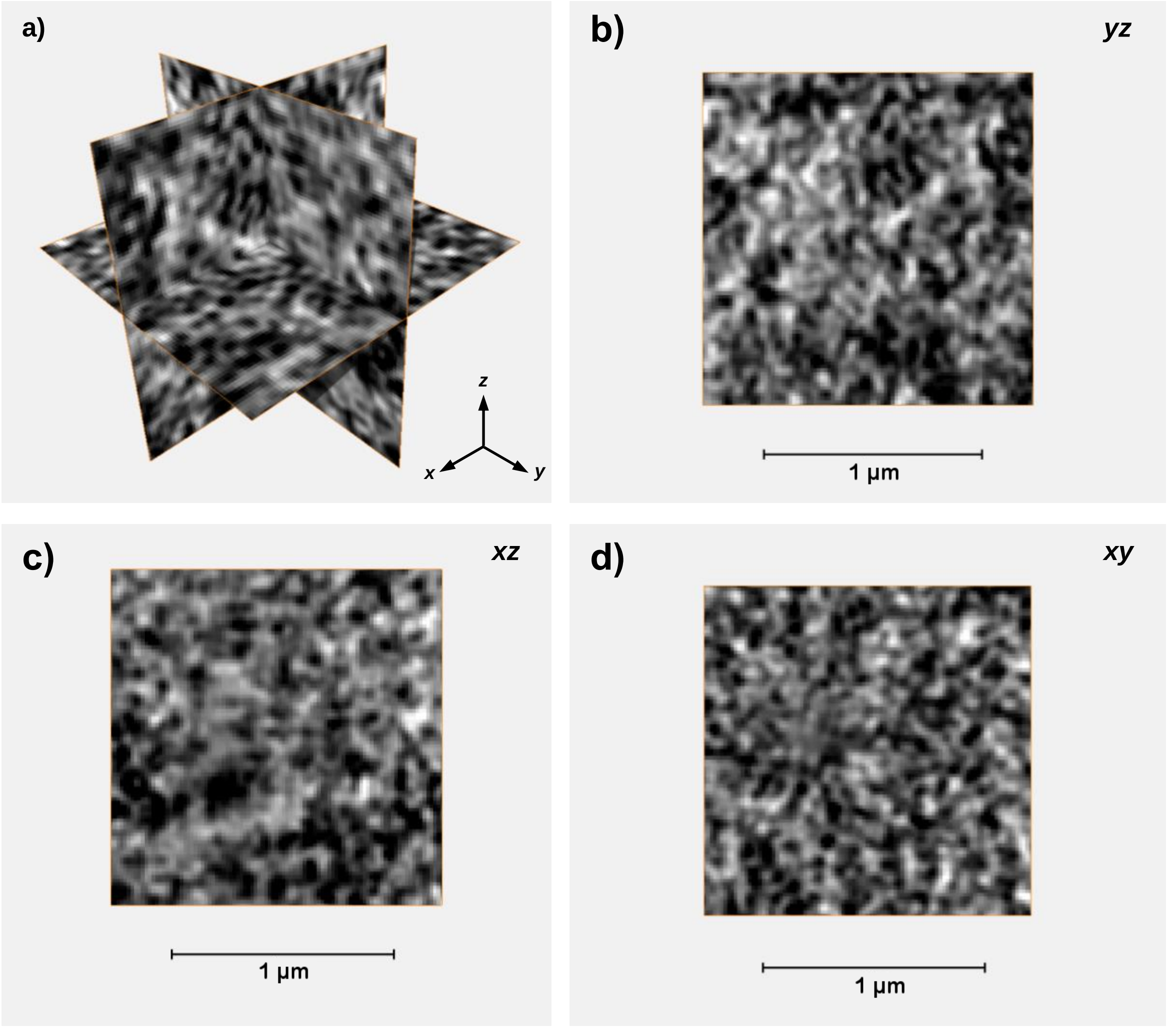

Figure S9. 3D nano-XRM images of the trypsin immobilized 3DOM hydrogels soaked in water. (a) Tomography slices (virtual slices) through intersecting orthogonal viewing planes, and (b-d) 2D view of the virtual slices through the imaging volume: (b) *yz* plane, (c) *xz* plane, and (d) *xy* plane.

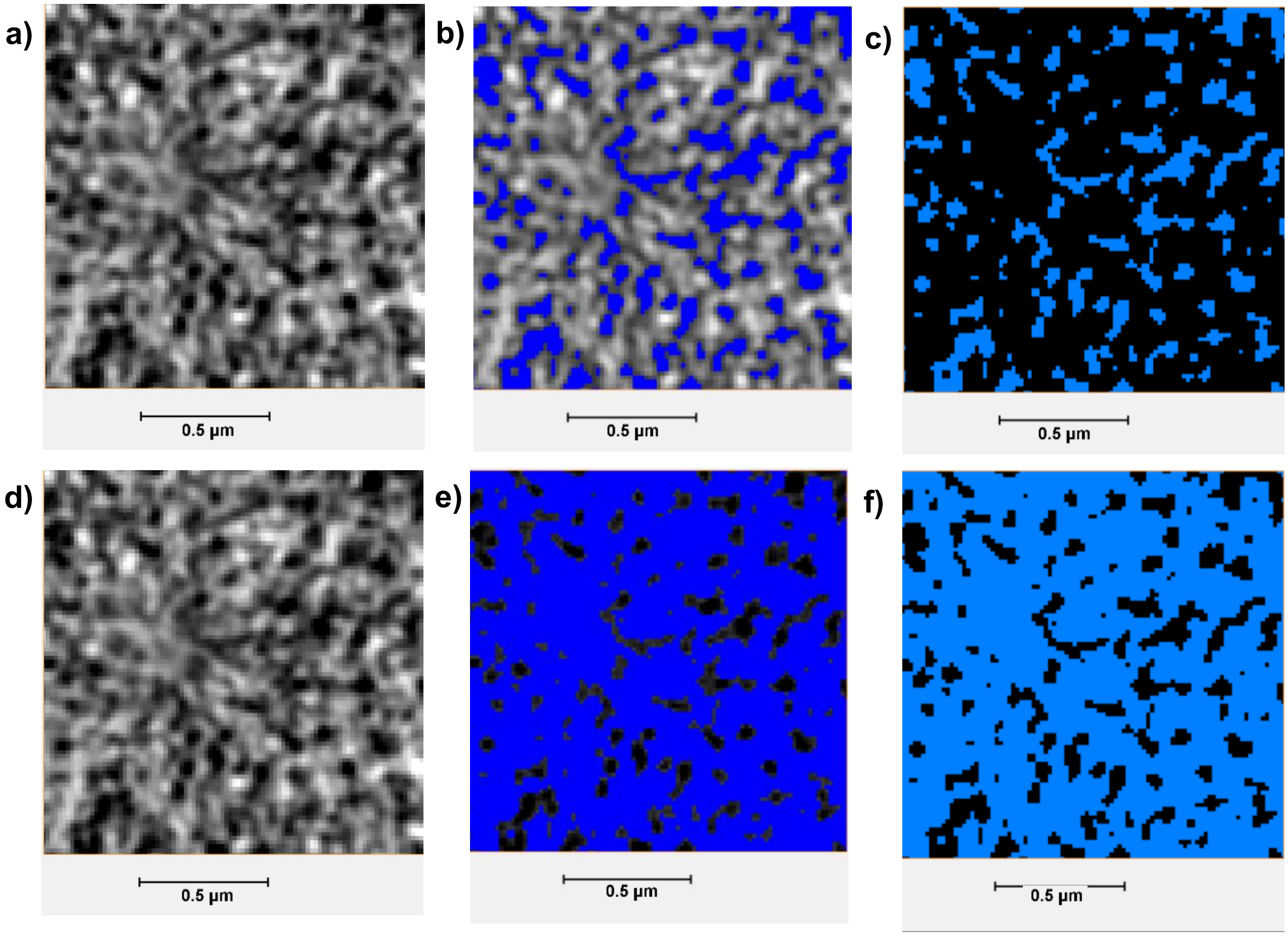

Figure S10. Segmentation of 3D nano-XRM images of the trypsin immobilized 3DOM hydrogels soaked in water. Segmentation of pore phase: (a) raw tomography slice in gray scale (dark phase corresponds to pores), (b) segmentation of pore phase using binary thresholding, and (c) binary image showing pore phase in blue. Segmentation of solid phase: (d) raw tomography slice in gray scale (dark phase corresponds to pores), (e) segmentation of solid phase using binary thresholding with the complementary intensity range as used for pore phase segmentation, and (f) binary image showing solid phase in blue.

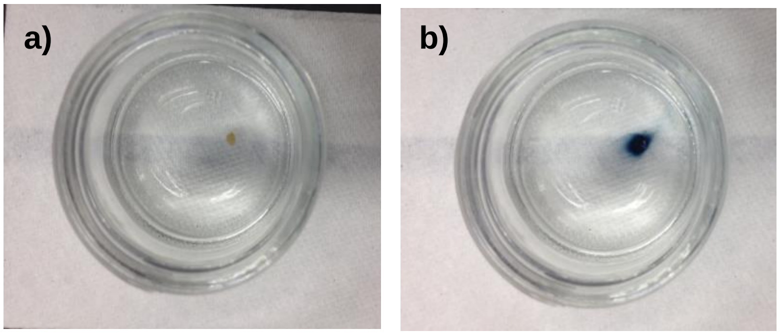

Figure S11. Photos of porous hydrogel-polyaniline at 0 min (a) and 10 min (b) after addition of ammonium persulfate (APS) to a mixture of 3DOM hydrogel, aniline, and water.

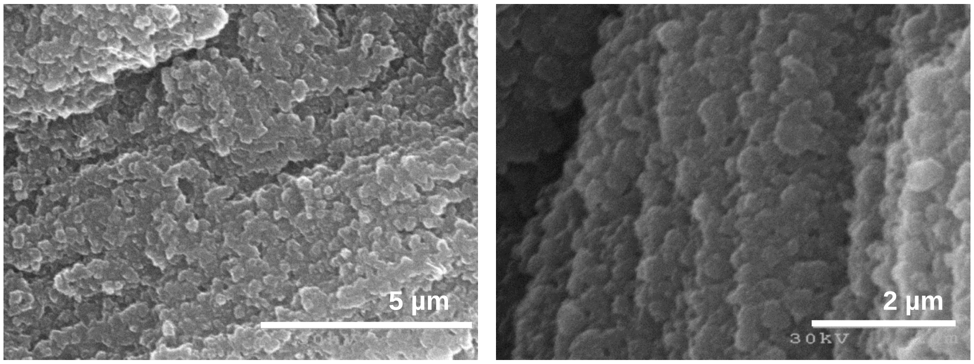

Figure S12. SEM images of the surfaces of 3DOM hydrogel/polyaniline composites.

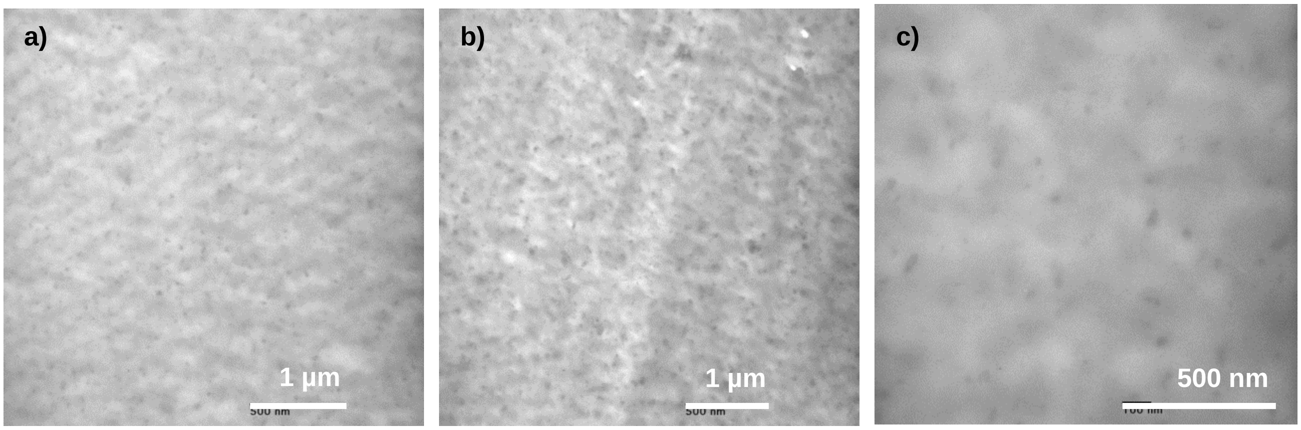

Figure S13. TEM images of the ~100 nm thin-section sample of 3DOM hydrogel/polyaniline composites.

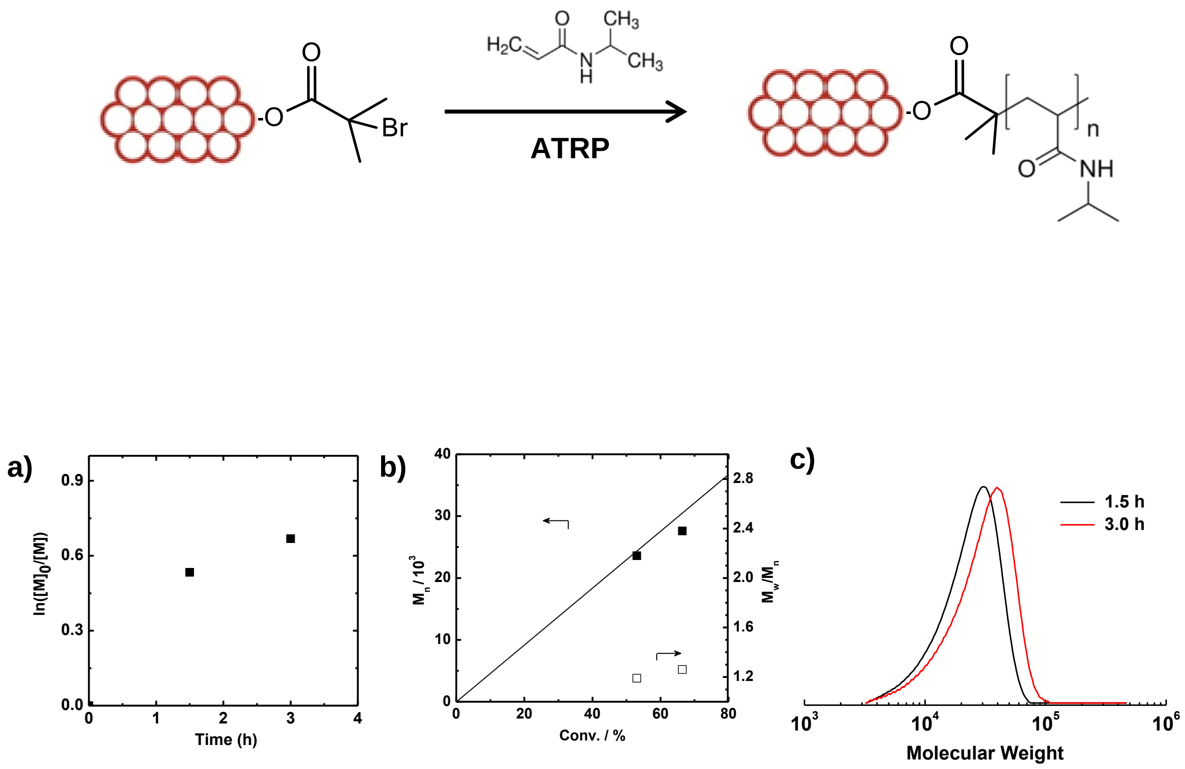

Figure S14. Kinetic plot of ln([M]$_0$/[M]) *vs* time (a), plot of M_n and M_w/M_n *vs* conversion (b), and GPC traces (c) for ATRP of *N*-isopropylacrylamide. Conditions: [*N*-isopropylacrylamide]$_0$/[EBiB]$_0$/[3DOM hydrogel-Br]$_0$/[Me$_6$TREN]$_0$/[CuCl]$_0$ = 4000/9/1/10/10, *N*-isopropylacrylamide/DMF = 1/1 (w/w), 25 °C.

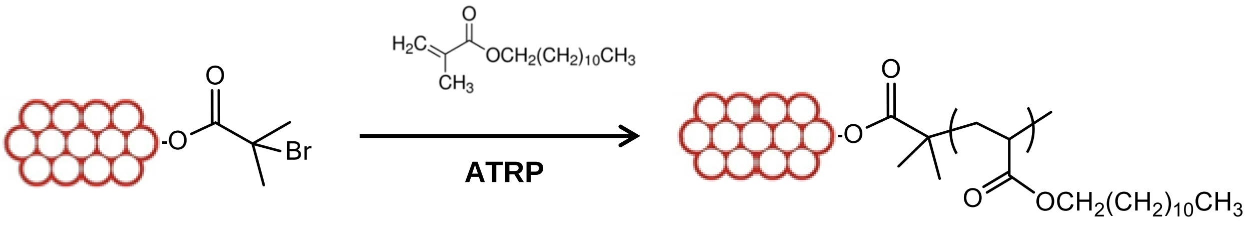

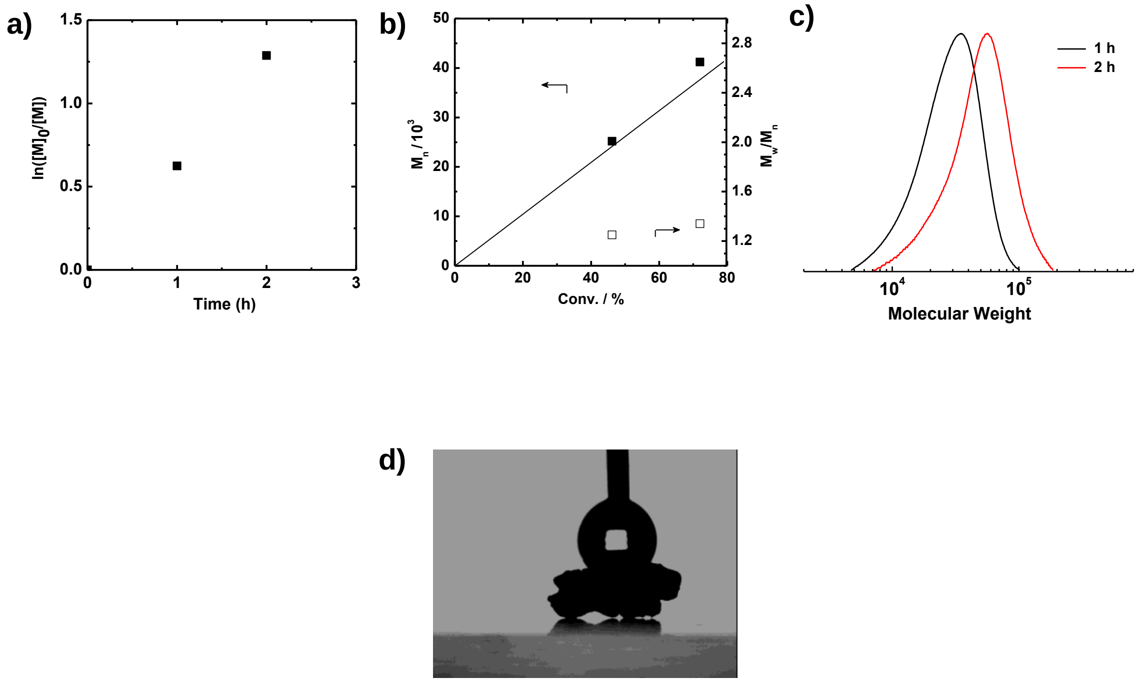

Figure S15. Kinetic plot of ln([M]$_0$/[M]) *vs* time (a), plot of M_n and M_w/M_n *vs* conversion (b), and GPC traces (c) for ATRP of lauryl methacrylate. Conditions: [lauryl methacrylate]$_0$/[EBiB]$_0$/[3DOM hydrogel-Br]$_0$/[PMDETA]$_0$/[CuBr]$_0$/[$CuBr_2$]$_0$ = 1000/4/1/5/4.5/0.5, lauryl methacrylate/DMF = 2/1 (v/v), 60 °C. (d) The change of hydrophilicity/hydrophobicity visualized by placing a drop of water on poly(lauryl methacrylate) modified 3DOM hydrogel (the water droplet stayed on top of the sample).

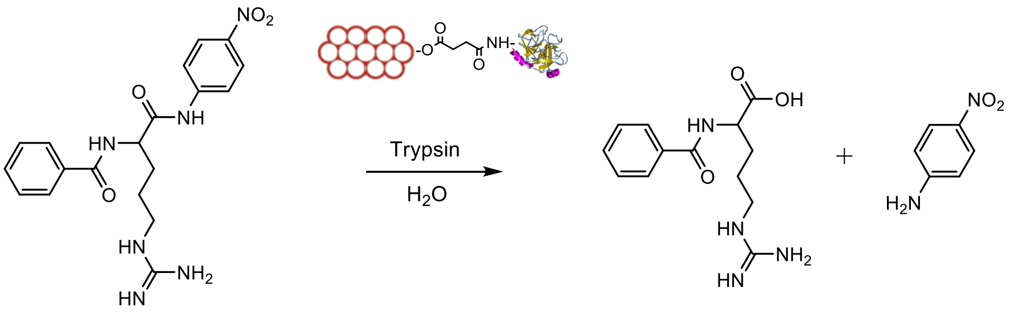

Scheme S4. Hydrolysis of *N*-α-benzoyl-L-arginine *p*-nitroanilide (BAPNA) by trypsin.

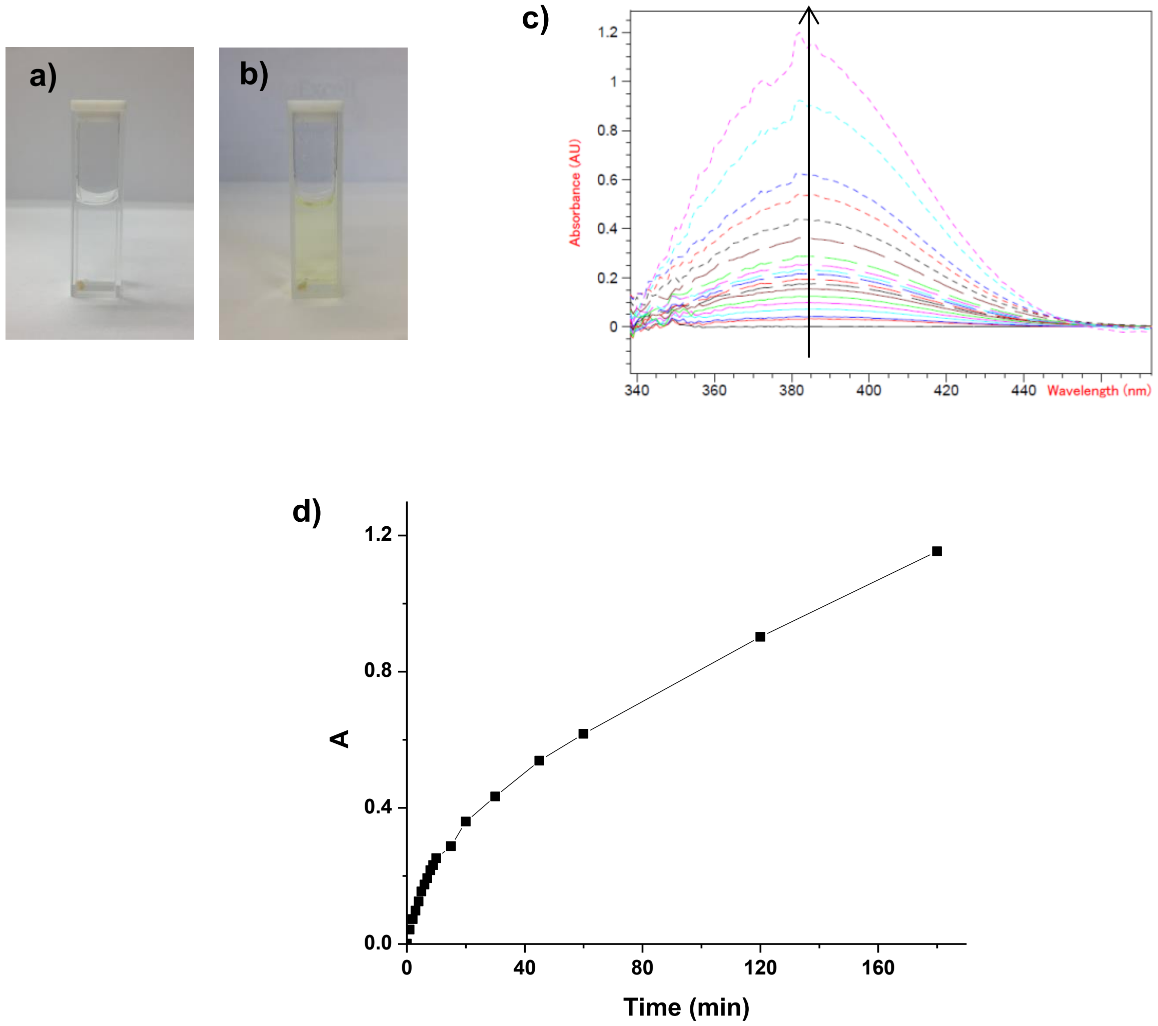

Figure S16. Photos of hydrogel-trypsin in BAPNA solution in a UV cuvette at (a) 0 h and (b) 3 h. (c) the UV-vis spectra from 0 to 3 h. (d) Plot of absorbance at 385 nm versus time for the UV-vis spectra in (c). Reaction conditions: 2 mM BAPNA solution was prepared by dissolving 4.4 mg of BAPNA in 0.1 mL of DMSO and diluted to 25 mL tris buffer (50 mM, pH 8). 0.2 mL of BAPNA solution (2 mM) was mixed with 2 mL of tris buffer (50 mM, pH 8) in a UV quartz cuvette, hydrogel-trypsin (0.5 mg) was added into the mixture and UV spectra were measured periodically.

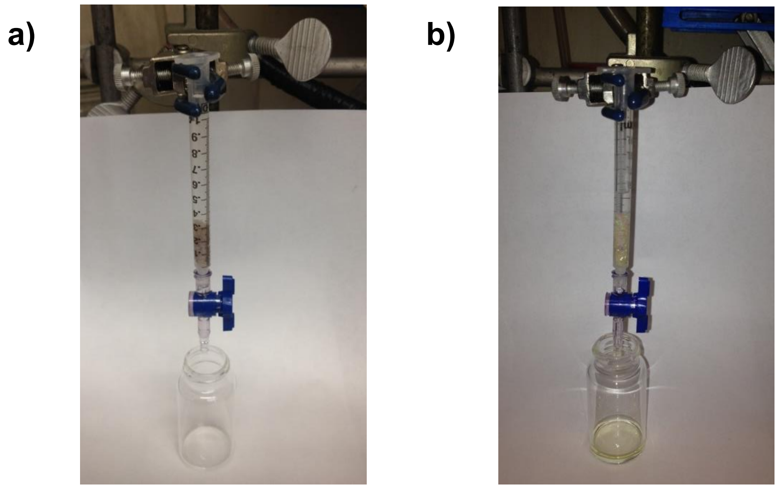

Figure S15. Photos of a syringe column filled with 3DOM hydrogel-trypsin before (a) and after (b) passing a solution of BAPNA through it. The images showed that as the BAPNA solution passed through the column and the color of the solution immediately changed from colorless to yellow, indicating that the hydrolysis of BAPNA by hydrogel-trypsin occurred in the column.

BCA Assay for protein quantification: A BCA protein assay kit was used to generate a calibration curve for the determination of residual trypsin concentration in the reaction mixture.[10] Pierce BCA protein assay reagent (bicinchoninic acid) was purchased from Thermo Fisher Scientific Inc. A trypsin stock solution (19.0 mg/mL) was prepared by dissolving 38.0 mg of trypsin, 40.8 mg of EDC·HCl, and 24.5 mg of NHS in 2.0 mL of TRIS buffer solution. The trypsin stock solution was diluted to 0.25, 0.125, 0.0625, 0.03125, 0.01563, and 0.00781 of its original concentration by adding TRIS buffer solution. 25 µL of every diluted sample were transferred into a 96 flat bottom transparent polystyrol well plate. BCA protein assay kit solutions B and A were mixed with a ratio of 1/50 (v/v). 200 µL of the

resulting BCA assay mixture was added to each sample in the wells. The samples were incubated at 37 °C for 30 min, and the absorption at 562 nm was measured using a TECAN infinite M1000 plate reader. The obtained calibration curve is shown in Figure S16.

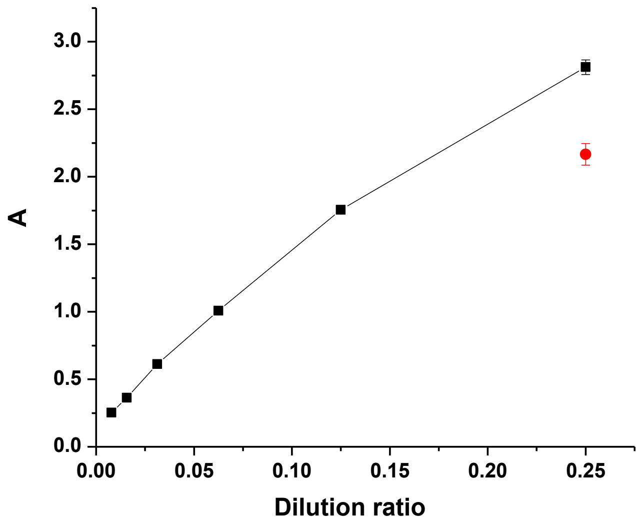

Figure S17. The calibration curve (black line) used for the determination of trypsin concentrations in the reaction solution. The red dot represented the sample prepared by diluting the final reaction mixture to 0.25 of its original concentration by adding TRIS buffer solution.

Then, 100 µL of the final reaction mixture was taken and diluted to 0.25 of its original concentration by adding TRIS buffer solution. 25 µL of the diluted sample was transferred into a 96 flat bottom transparent polystyrol well plate. BCA protein assay kit solutions B and A were mixed in a ratio of 1/50 (v/v). 200 µL of the resulting BCA assay mixture was added to the sample in the well. The samples were incubated at 37 °C for 30 min, and the absorption at 562 nm was measured using a TECAN infinite M1000 plate reader. The concentration of trypsin in the final reaction mixture was 14.63 mg/mL according to the calibration curve shown in Figure S16. The concentration of trypsin in the original reaction mixture (2.0 mL) was 19.0 mg/mL, so the amount of trypsin on the hydrogel was 8.74 mg for 38 mg of hydrogel, or 18.7 wt%.

For the determination of trypsin concentration in the solution, a BCA protein assay kit was used to generate a calibration curve. A trypsin stock solution (2.0 mg/mL) was prepared by dissolving 20.0 mg

of trypsin in 10.0 mL of TRIS buffer solution. The calibration curve was measured using the trypsin concentrations outlined in Table 1. 25 µL of every sample were transferred into a 96 flat bottom transparent polystyrol well plate. BCA protein assay kit solutions B and A were mixed in a ratio of 1/50 (v/v). 200 µL of the resulting BCA assay mixture was added to each sample in the wells. The samples were incubated at 37 °C for 30 min, and the absorption at 562 nm was measured using a TECAN infinite M1000 plate reader. The obtained calibration curve is shown in Figure S17.

Trypsin concentration (µg/mL)	0	25	50	75	100	250
TRIS buffer solution (µL)	1000	987.5	975	962.5	950	875
Trypsin stock solution (µL)	0	12.5	25	37.5	50	125

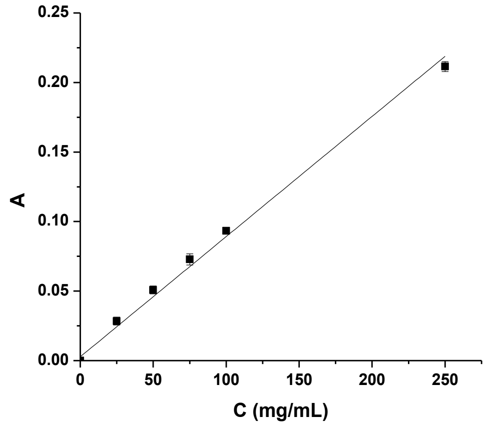

Figure S19. Calibration curve for the determination of trypsin concentrations in the performed leaching experiment.

Leaching experiment: The sample hydrogel-trypsin was suspended in TRIS buffer solution for 1 d. The supernatant was measured by BCA protein assay and no absorption at 562 nm was observed, indicating that no trypsin leaching had occurred. A previously acquired calibration curve (Figure S17) was used to quantify the amount of enzyme leached out the sample.

Trypsin activity determination: The activity of trypsin and hydrogel supported trypsin (hydrogel-trypsin) was quantified by the release of *N*-α-*p*-tosyl-L-arginine from the substrate *N*-α-*p*-tosyl-L-arginine methyl ester hydrochloride (TAME) according to previously reported procedures.[10-11] The

reaction was monitored photometrically through the measurement of the extinction coefficient at 247 nm. In the following discussion, one unit is defined as the amount of enzyme releasing 1 µmol *N*-α-*p*-tosyl-L-arginine per minute under the denoted conditions.

A TAME stock solution was prepared (189 mg TAME in 50 mL water). The enzyme is dissolved in HCl aqueous solution (1 mM) with an enzyme concentration of 5.5 µg/mL. For the activity measurements, a 1.5 mL quartz cuvette with 75 µL TAME stock solution, 200 µL enzyme solution (1.1 µg enzyme), and 1225 µL Tris buffer was prepared. The obtained curve was shown in Figure S18. The resulting slope of the linear regression is 0.0155 min^{-1}. By using equation 1, the activity of natural trypsin was calculated to be 587.1 units.

For the evaluation of the activity of the hydrogel-trypsin sample, 0.6 mg of the sample containing 6.6 µg of enzyme (data from BCA assay) was added to a mixture of 75 µl TAME stock solution, 100 µL HCl aqueous solution (1 mM), and 1325 µL Tris buffer. The extinction at 247 nm was acquired at timed intervals. Between each acquisition, the suspension was homogenized by shaking. The obtained curve is shown in Figure S19. The resulting slope of the linear regression is 0.06553 min^{-1}, which corresponds to 24.3 units per mg of enzyme for hydrogel-trypsin.

The enzyme activity was determined from the slope of the obtained linear curve according to eq. 1.

$$\text{Enzyme activity} = \frac{d[E_{247}]/dt}{0.54 \times W_0} \times 1.5 \qquad \text{(eq. 1)}$$

where E_{247} is the extinction at 247 nm, t is the reaction time, 0.54 is the extinction of 1 µmol *N*-α-*p*-tosyl-L-arginine, W_0 is the initial weight of enzyme in mg per 0.1 mL solution, 1.5 is the total volume of sample in mL.

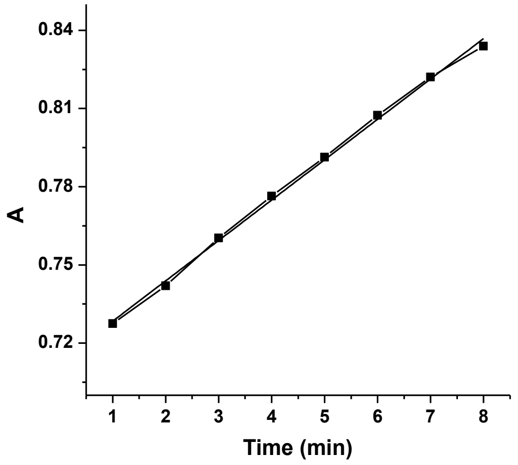

Figure S20. Determination of the activity of trypsin. Equation: $y = 0.71289 + 0.0155x$, Adj. R-Square: 0.99771. 0.0155*1.5/0.54/0.0011 *15 = 587.1 unit

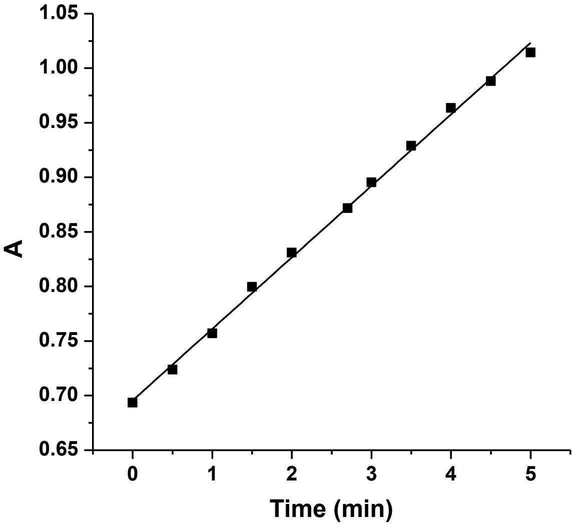

Figure S21. Determination of the activity of hydrogel-trypsin. Equation: $y = 0.69564 + 0.06553x$, Adj. R-Square: 0.99775. Dried 0.6 mg of hydrogel-trypsin (trypsin content: 18.7 wt%) containing 0.1122 mg trypsin, 0.06553*1.5/0.54/0.1122 *15 = 24.3 unit.

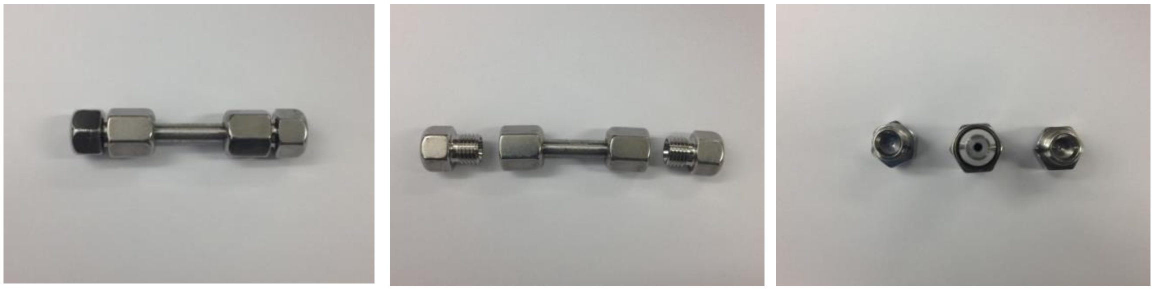

Figure S22. HPLC column used for the preparation of the trypsin-immobilized column. Varian Pursuit Ultra 2.4 C18, length: 50 mm, inner diameter: 2.0 mm.

Determination of the activity of immobilized trypsin in a HPLC column: The activity of the immobilized trypsin was estimated by an off-line method using *N*-α-Benzoyl-L-arginine ethyl ester hydrochloride (BAEE) as a standard substrate.[12] BAEE is a non-chromogenic ester that can be hydrolyzed by trypsin to generate *N*-benzoyl-L-arginine (BA). The substrate and product have similar UV–vis absorption profiles, except for a narrow wavelength window (253–255 nm) where the product is more absorptive. Therefore, a chromatographic method was required for the separation of BAEE and BA.

The procedures for the determination of the activity of trypsin-immobilized in an HPLC column include two steps:

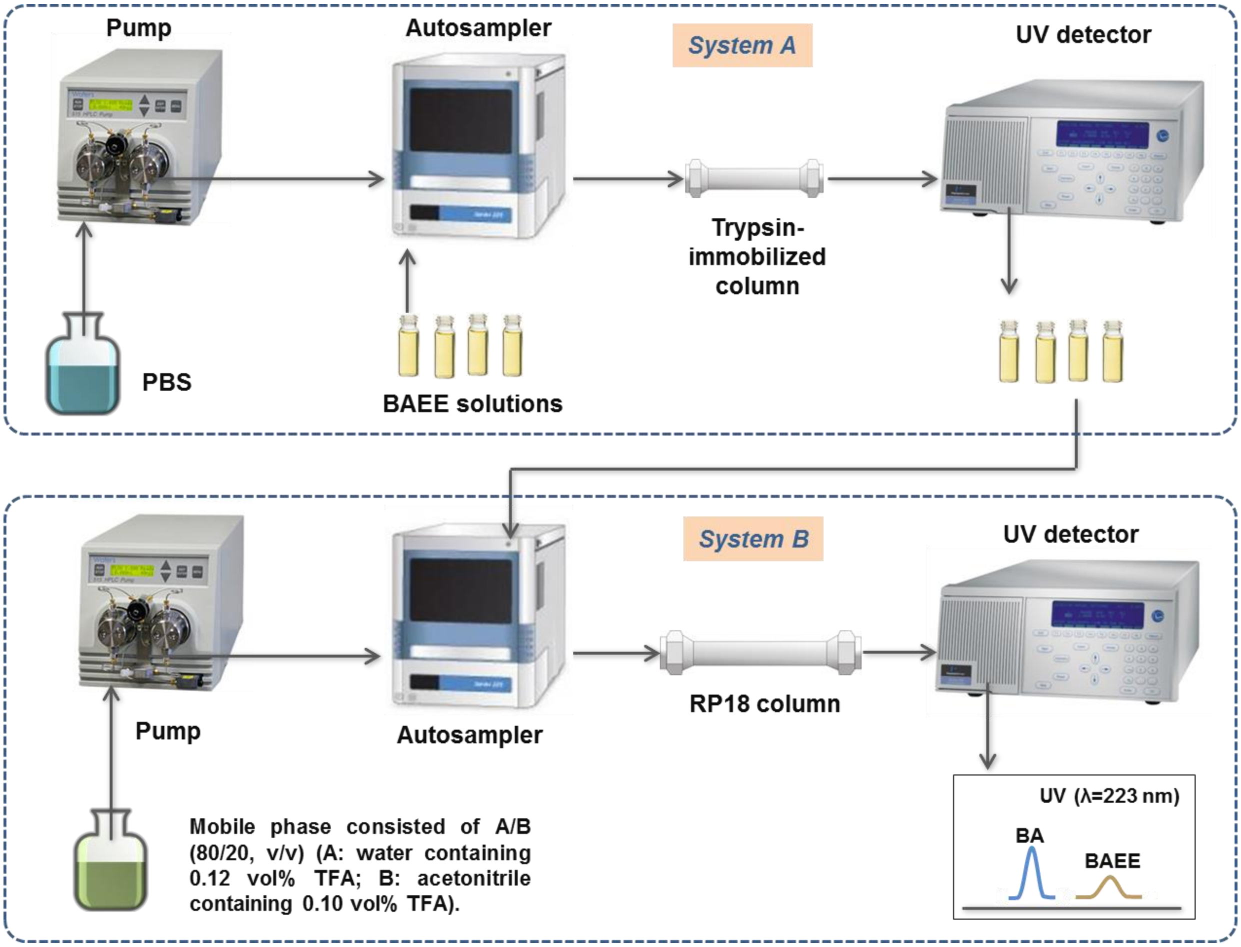

Figure S23. Determination of the activity of trypsin-immobilized in an HPLC column. Instrumentation: Waters 515 HPLC pump, Perkin Elmer Series 200 UV/Vis detector, Perkin Elmer 225 autosampler, the RP18 column used was Luna 3 μm C18(2) 100 Å, LC Column 50 × 2.00 mm, 3 micron.

Step 1 (trypsin-immobilized column, system A): The column containing the trypsin-immobilized hydrogel was equilibrated for 30 min with phosphate buffered saline (PBS, 50 mM, pH 8) at 37 ^{o}C. Aliquots of 40 μL BAEE solutions with increasing concentration (5–200 mM in PBS) were injected to the column containing the trypsin-immobilized hydrogel at a flow rate of 0.02 mL/min. The eluent was monitored at 223 nm by a UV/Vis detector. The species eluted from the column containing the trypsin-immobilized hydrogel were contained in the first 1.5 mL eluent (75 min elution time). As shown in Figure 4, the peaks indicated the elution of BAEE and its hydrolyzed products through the column, and the intensity of the peak increased with the increase of the concentration of the BAEE solutions.

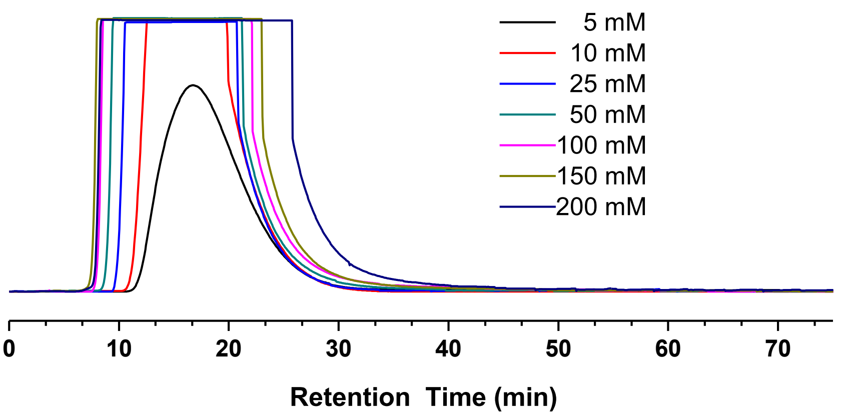

Figure S24. On-line monitoring of the absorption at 223 nm versus retention time for BAEE solutions with various concentrations of BAEE after passing through the column containing the trypsin-immobilized hydrogel.

Step 2 (RP18 column, system B): The 1.5 mL collected solution for each sample was injected off-line in system B. The collected solutions contained the product BA and unreacted substrate BAEE.

The peak at 6-8 min retention time was from unreacted BAEE, and the peak at 3.5-4 min retention time was from BA, the product from hydrolysis of BAEE. When 200 or 150 mM BAEE substrate solution was used, BAEE could be partially hydrolyzed by the trypsin immobilized on the hydrogels. When 50 mM or less BAEE substrate solution was used, BAEE was completely hydrolyzed by the trypsin immobilized on the hydrogels.

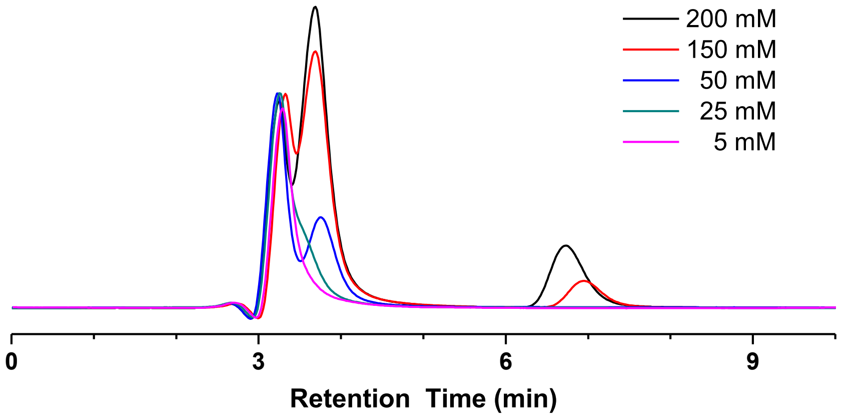

Figure S25. On-line monitoring of the absorption at 223 nm versus retention time for the collected solutions from step 1 after passing through a RP18 column. In system B, the mobile phase consisted of A/B (80/20, v/v) (A: water containing 0.12 vol% TFA; B: acetonitrile containing 0.10 vol% TFA). The flow rate was 0.1 mL/min and the detector was set at 223 nm, the column temperature was 25 ◦C.

A calibration curve was also made by injecting BAEE solutions with various concentrations of BAEE into system B, and then plotting the integrated UV responsive peak areas to the concentration of BAEE in each solution. A linear regression was obtained with R^2 = 0.99977

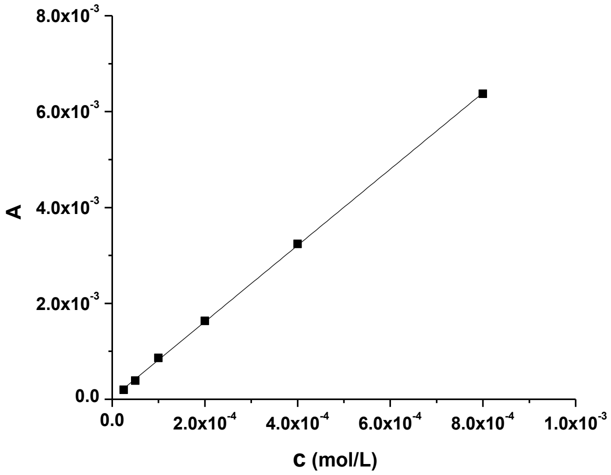

Figure S26. Calibration curve of the integrated UV responsive peak areas versus the concentration of BAEE in each solution.

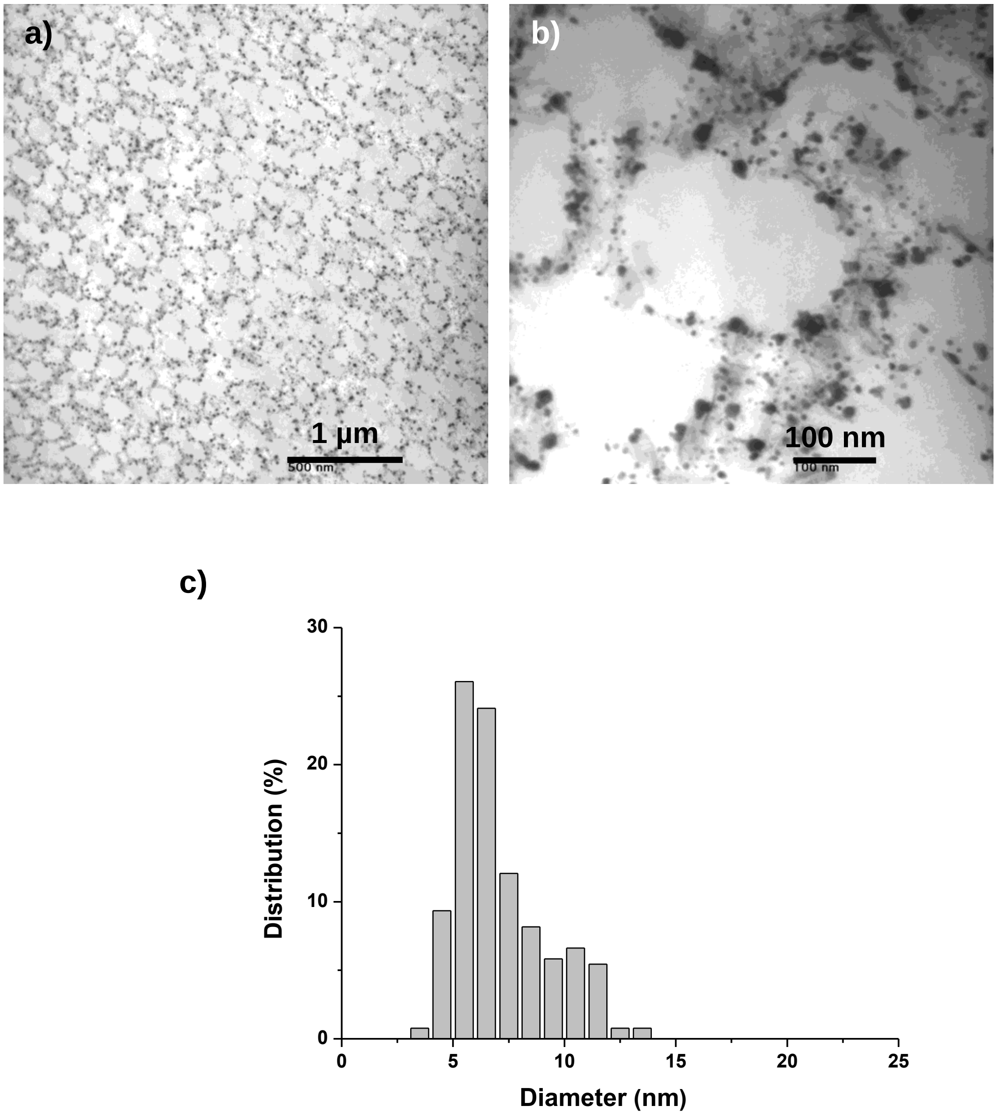

Figure S27. Additional TEM images (a,b) and size distribution analysis (c) of the ~100 nm thin-section sample of 3DOM hydrogel-Au NPs composite. The average diameter of the Au nanoparticles was 7.1 nm.

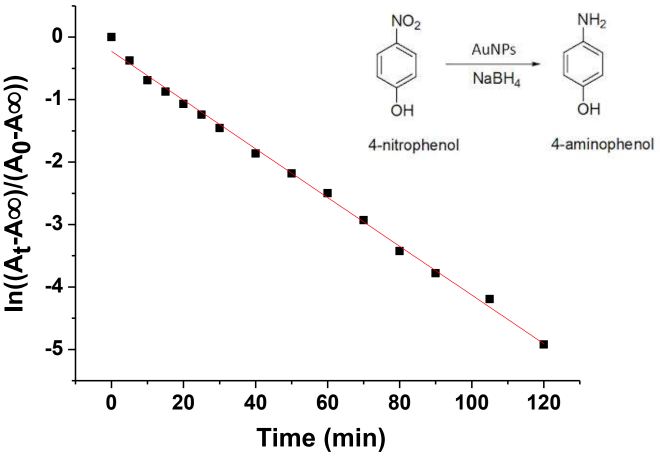

Figure S28. The linear fit of the experimental results to first-order analysis according to eq. 1 for the successive UV-vis spectra for the catalytic reduction of 4-nitrophenol into 4-aminophenol by Au NPs loaded 3DOM hydrogels. Adj. R-square: 0.99626, Slope: -0.03902 min^{-1}.

The catalysis activity of the Au NPs loaded 3DOM hydrogels was measured by using a model reaction of the catalytic reduction of 4-nitrophenol into 4-aminophenol with $NaBH_4$. The UV-vis spectroscopy was used to monitor the reduction process, and the results are shown in Figure 5d. The characteristic absorption peak of 4-nitrophenol located at 400 nm decreased quickly upon the addition of the catalysts to the reaction mixture. Meanwhile, a new peak at 290 nm appeared that was attributed to the formation of 4-aminophenol. Since the concentration of $NaBH_4$ greatly exceeded 4-nitrophenol and the catalyst and remained essentially constant during the reduction, the kinetics of this reduction was supposed to follow pseudo-first-order to the concentration of 4-nitrophenol, and the kinetic equation can be defined by eq. 2:

$$In\left(\frac{A_t - A_\infty}{A_0 - A_\infty}\right) = -k_{ap}t \qquad \text{(eq. 2)}$$

where t is the reaction time, A_0 is the initial absorbance at time zero, A_t is the absorbance at time t, A_∞ is the absorbance when the reaction is completed, and k_{ap} is the apparent rate constant.[13] As seen in Figure S26, the linear-fit plot coincides with eq. 2, indicating pseudo-first-order kinetics for this reduction. The values of $k_{ap} = 6.5\ 10^{-4}\ s^{-1}$, which was comparable with, or higher than, some other types of supported metal nanocatalysts, such as dendrimer-metal nanocomposites ($10^{-5} \sim 10^{-1}\ s^{-1}$)[13-14] and SiO_2-Pt nanohybrids ($\sim 10^{-3}\ s^{-1}$).[15] The high values determined for k_{ap} in the successive cycles of catalytic reduction demonstrated the high catalytic activity of Au NPs loaded 3DOM hydrogels.

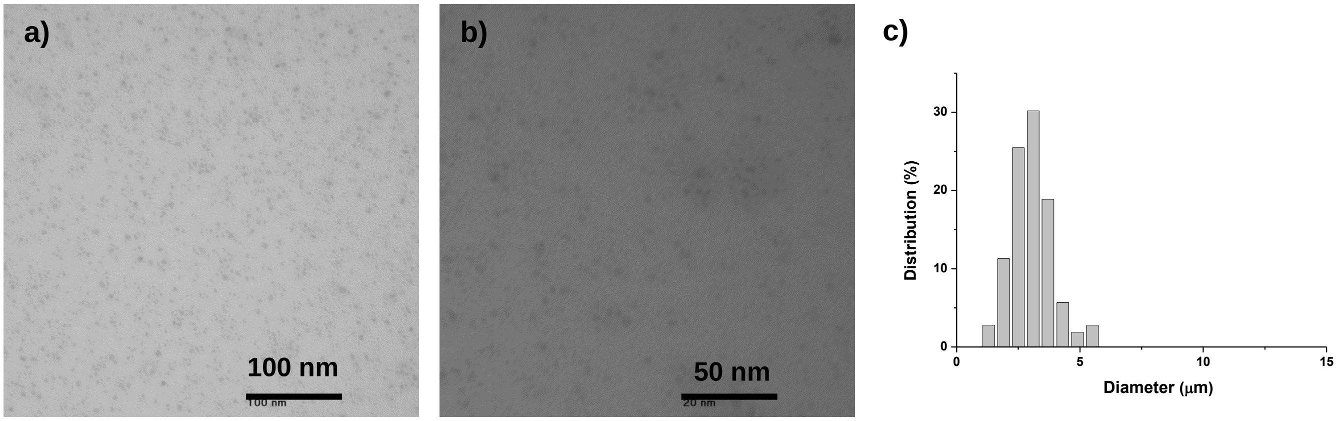

Figure S29. TEM images (a,b) and size distribution analysis (c) of Fe_3O_4 nanoparticles. The average diameter of Fe_3O_4 nanoparticles was 3.1 nm.

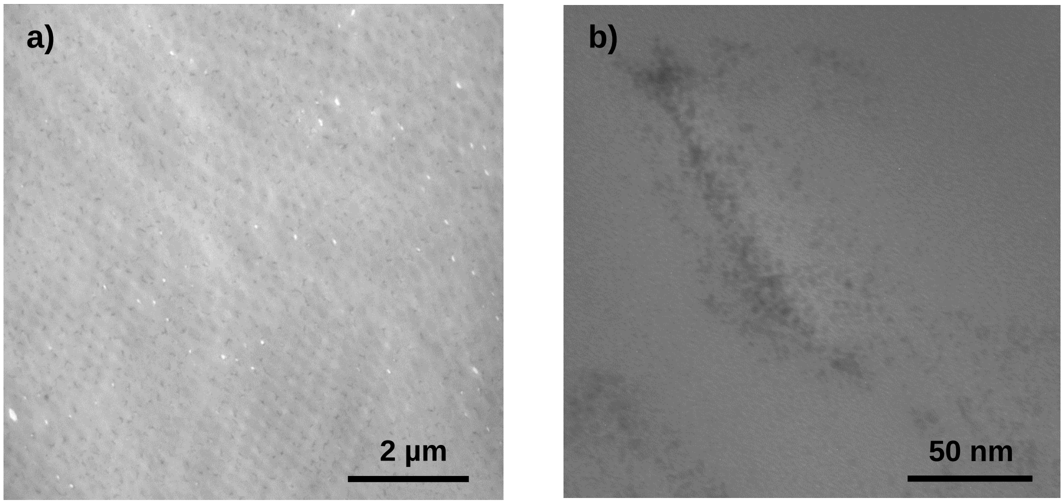

Figure S30. Additional TEM images of the ~100 nm thin-section sample of 3DOM hydrogel-Fe_3O_4 nanoparticles composite.

Supplementary Movies

Movie S1. Movie showing the porous network through a cropped reconstructed volume of 3D nano-XRM image of the 3DOM hydrogels shown in Figure 2a.

Movie S2. Movie showing virtual slices (tomography slices) through the reconstructed volume of the 3D nano-XRM image of the 3DOM hydrogels shown in Figure 2a.

Movie S3. Movie showing the volume rendering of the pore phase in the cropped volume of reconstructed 3D nano-XRM image of the 3DOM hydrogels shown in Figure 2b: (left) without transparency, and (right) with increased transparency.

Supplementary References

1. He, H. K.; Li, W. W.; Zhong, M. J.; Konkolewicz, D.; Wu, D. C.; Yaccato, K.; Rappold, T.; Sugar, G.; David, N. E.; Matyjaszewski, K. *Energy Environ. Sci.* **2013,** *6*, 488.
2. Bencherif, S. A.; Siegwart, D. J.; Srinivasan, A.; Horkay, F.; Hollinger, J. O.; Washburn, N. R.; Matyjaszewski, K. *Biomaterials* **2009,** *30*, 5270-5278.
3. Zou, D.; Ma, S.; Guan, R.; Park, M.; Sun, L.; Aklonis, J. J.; Salovey, R. *J. Polym. Sci., Part B: Polym. Phys.* **1992,** *30*, 137-144.
4. Wang, Z.; Kiesel, E. R.; Stein, A. *J. Mater. Chem.* **2008,** *18*, 2194-2200.
5. Ding, H.; Wan, M.; Wei, Y. *Adv. Mater.* **2007,** *19*, 465-469.
6. Ge, J.; Hu, Y.; Biasini, M.; Dong, C.; Guo, J.; Beyermann, W. P.; Yin, Y. *Chem.-Eur. J.* **2007,** *13*, 7153-7161.
7. Xia, Y.; Yin, X. C.; Burke, N. A. D.; Stover, H. D. H. *Macromolecules* **2005,** *38*, 5937-5943.
8. Beers, K. L.; Matyjaszewski, K. *J. Macromol. Sci., Pure Appl. Chem.* **2001,** *38*, 731-739.
9. Dutertre, F.; Pennarun, P.-Y.; Colombani, O.; Nicol, E. *Eur. Polym. J.* **2011,** *47*, 343-351.
10. Schlossbauer, A.; Schaffert, D.; Kecht, J.; Wagner, E.; Bein, T. *J. Am. Chem. Soc.* **2008,** *130*, 12558-12559.
11. Hummel, B. C. W. *Can J Biochem Physiol.* **1959,** *37*, 1393-1399.
12. Calleria, E.; Temporinia, C.; Gasparrinib, F.; Simoneb, P.; Villanib, C.; Cioglib, A.; Massolinia, G. *J. Chromatogr. A* **2011,** *1218*, 8937-8945.
13. Esumi, K.; Isono, R.; Yoshimura, T. *Langmuir* **2004,** *20*, 237-243.
14. Esumi, K.; Miyamoto, K.; Yoshimura, T. *J. Colloid Interface Sci.* **2002,** *254*, 402-405.
15. Zhou, L.; Gao, C.; Xu, W. *Langmuir* **2010,** *26*, 11217-11225.
